# A nano-immuno-cruise delivery system encapsulated lipid-integrated bilayer ameliorate acute lung injury by interfering neutrophil infiltration

**DOI:** 10.1016/j.mtbio.2025.102563

**Published:** 2025-11-17

**Authors:** Guiquan Liu, Xinting Wang, Jia Liu, Haonan Wu, John Osilama Thomas, Yan Zhu, Xi Wang, Jian Yang

**Affiliations:** a State Key Laboratory of Chinese Medicine Modernization, Tianjin University of Traditional Chinese Medicine, Tianjin, 301617, China; b Institute of Traditional Chinese Medicine, Tianjin University of Traditional Chinese Medicine, Tianjin, 301617, China; c Haihe Laboratory of Modern Chinese Medicine, Tianjin, 301617, China; d Department of Orthopedics, Binhai New Area Hospital of Traditional Chinese Medicine and the Fourth Affiliated Hospital of Tianjin University of Traditional Chinese Medicine, Tianjin, 301617, China

**Keywords:** Neutrophil membrane, Acute lung injury (ALI), Platelet-neutrophil aggregates, ROS-triggered drug release, Drug delivery system

## Abstract

Effective therapies for acute lung injury (ALI) are still lacking due to poor drug targeting and accumulation, inability to surmount the lung barrier. Nanosystem camouflaged with the membrane of immune cell and responsive lipids offers potential solution. In this study, we developed a nano-immuno-cruise drug delivery system (DDS) using the anti-inflammatory drug naringin (Nar) loaded reactive oxygen species (ROS)-responsive liposomes fused with activated neutrophil membranes (TK-NLP). The TK-NLP showed great targeting ability in both injured 2D epithelial cell model and 3D ALI model based on a 3D printed mimicking lung organ (mLO) with a dynamic environment on chip. Interestingly, TK-NLP could effectively inhibit the formation of platelet-neutrophil aggregates (PNAs), thereby showing great potential for suppressing PNA-mediated inflammatory cascades. Subsequently, in the mouse model of ALI, TK-NLP aggregate specifically at pneumonia sites and respond to the overexpressed ROS with the release of Nar, which reduced neutrophil infiltration and inflammatory factors secretion, protecting the integrity of the lung barrier to ameliorate ALI. Collectively, this nano-immuno-cruise DDS integrating immune camouflage, ROS-responsive controlled drug release, and targeted interference with PNAs formation, offers a novel and promising strategy for precision therapy and barrier repairment for ALI.

## Graphical abstract

**Figure:**
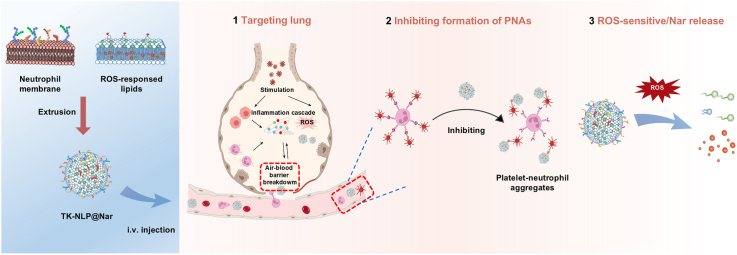

## 1 Introduction

Acute lung injury (ALI) is a severe clinical condition characterized by diffuse inflammation of the lung parenchyma and persistent hypoxemia. It has a rapid onset and a high mortality rate of 30–40 %, posing a significant threat to patient's survival and quality of life [[1], [2], [3]]. As the first line of defense in the immune system, neutrophils play a crucial role in the onset and progression of ALI, they rapidly migrate to sites of inflammation and eliminate pathogens by releasing reactive oxygen species (ROS), neutrophil extracellular traps (NETs) and granule-derived enzymes [[4], [5], [6]]. However, excessive neutrophil activation can result in tissue damage and exacerbate inflammation [7,8]. Recently, studies have shown that activated platelets interact with neutrophils through receptor-ligand binding (P-selectin and PSGL-1), promoting neutrophil rolling, migration, and NETs formation [9,10]. Moreover, the formation of platelet-neutrophil aggregates (PNAs) acts as a potent amplifier of the inflammatory cascade, intensifying pulmonary inflammation and tissue injury [11,12]. Therefore, the development of delivery systems capable of targeting the pulmonary inflammatory microenvironment and effectively disrupting the formation of PNAs represents a promising therapeutic strategy to attenuate the progression of ALI.

ROS, a byproduct of oxidative cellular metabolism, plays a crucial role in regulating biological functions and contributing to pathological conditions such as cancer and inflammation when overproduced [13,14]. Leveraging this phenomenon, ROS-responsive DDSs have gained prominence as they selectively release drug payloads in ROS-rich environments [15]. These DDSs incorporate ROS-responsive substances such as thione or diselenide bonds into nanoparticles that respond to ROS or H_2_O_2_, by rapidly releasing the drug payload [16,17]. For example, Fan et al. synthesized ROS-responsive red light carbon dots TK-methylprednisolone nanoparticles for ALI treatment, which successfully targeted ROS-damaged tissues to alleviate lung injury [18]. Similarly, Guo et al. designed ROS-sensitive polymers to encapsulate Cu, demonstrating effective drug release and cell death in cancer cells under high ROS conditions [19]. Such systems provide preciseand ROS-triggered drug release, achieving “on-demand” delivery and minimizing side effects in healthy tissues [20]. However, ROS-responsive nanoparticles provide conditional drug release at lesion sites, sole reliance on passive targeting mechanisms presents challenges, such as insufficient drug accumulation at target sites and difficult in overcoming the physiological barrier of the lungs [21,22]. Furthermore, uncertainties remain regarding whether the time window for ROS-triggered drug release aligns with therapeutic requirements. To address these issues, cell membrane camouflage technology has emerged as a promising strategy for enhancing precise targeting and drug release efficiency [23,24].

Liposomes are considered a leading platform among nanocarriers, distinguished by their superior biocompatibility, biodegradability, and FDA-approved status for clinical applications [25,26]. Their phospholipid bilayer structure, which is similar to that of cell membranes, allows for the embedding of the neutrophil membrane with the liposome, thereby minimizing its coverage over the core nanoparticle [27]. Neutrophil membranes excel in targeting inflammation due to their chemotactic ability, can evade the immune system and extending circulation time, traverse the pulmonary barrier, and localize to inflammatory sites via receptor interactions, qualities that make them an ideal carrier for inflammation-related disease therapy [30,31]. Neutrophil membranes in the nano-immuno-navigation system show matched targeting, immune evasion, and biocompatibility compared to macrophage membranes, and demonstrates superior ability to cross physiological barriers [28,29]. Cell membrane materials hardly controlled release drug, while fused with ROS responsive LP, this composite system simultaneously possesses all the functions of neutrophil membranes and controlled drug release capability [32,33].

**Scheme 1:**
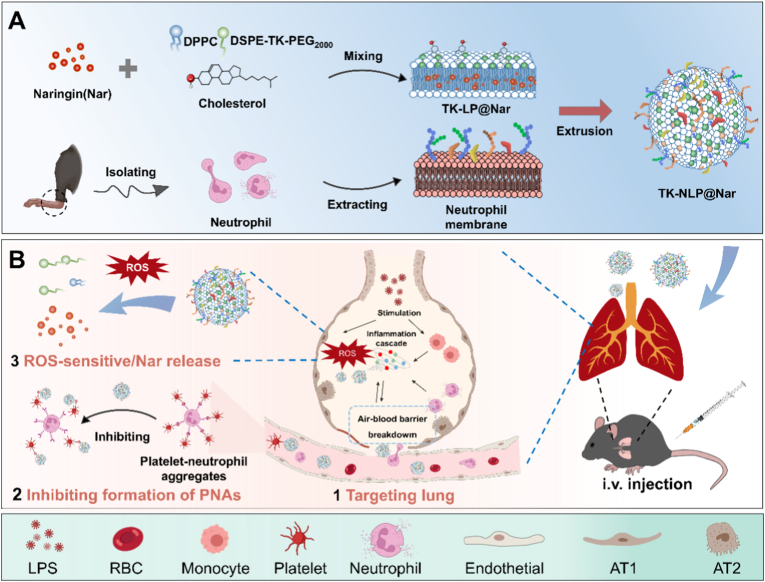
Schematic illustration of the preparation of TK-NLP@Nar and the treatment of ALI. (A) The preparation process of TK-NLP@Nar. (B) After intravenous injection in an ALI mouse, TK-NLP@Nar effectively accumulated in the lung lesion site, released Nar triggered by ROS, and ameliorated ALI by protecting the lung barrier, inhibiting the release of inflammatory factors and PNAs formation.

Naringin (Nar), a natural flavonoid abundantly in citrus fruits, has emerged as a promising candidate for treating inflammatory lung diseases due to its potent anti-oxidant and anti-inflammatory properties [34,35]. However, its therapeutic potential is severely limited by poor aqueous solubility and low bioavailability, common challenges for natural compounds [36]. To address these limitations, we designed a Nar-loaded, ROS-responsive liposome embedded with activated neutrophil membranes (TK-NLP@Nar), integrating active targeting of injured lung sites with ROS-responsive controlled drug release (Scheme 1). The modified neutrophil membranes enriched with functional proteins could target activated platelets and reduce the formation of PNAs. In addition, neutrophil membranes enable the liposomes to target injured 2D cell models and 3D ALI models based on a mimicking lung organ (mLO) cultured in both static and dynamic chip environments. TK-NLP@Nar effectively targeted and accumulated in the site of pneumonia, responding to ROS to mediate controlled Nar release, which effectively reduced the release of inflammatory factors and neutrophil infiltration, protected lung barrier integrity to ameliorate ALI. By concurrently achieving targeted delivery to inflammatory sites, inhibition of PNAs formation, and ROS-responsive drug release, TK-NLP@Nar offers a unique multi-mechanistic therapeutic strategy for ALI.

## 2 Materials and methods

### 2.1 Materials

Dichloromethane (CH_2_Cl_2_, D116146) and methanol (MeOH, M116118) were obtained from Shanghai Aladdin Biochemical Technology Co., Ltd (Shanghai, China). DSPE-PEG_2000_ (YS-DM2301) and DSPE-TK-PEG_2000_ (YS-DTKM2301) were obtained from Xi'an Ruixi Biological Technology Co., Ltd. (Xian, China). IL-6 ELISA Kit (431304), IL-1β ELISA Kit (432604), TNF-α ELISA Kit (430904), and APC-anti-mouse Ly6G Antibody (127613) were obtained from BioLegend, Inc. (San Diego, CA). Anti-ZO-1 antibody (ab98687), anti-CXCR2 antibody (ab65968), anti-E-cadherin antibody (ab231303), anti-IL-1R antibody (ab212208), anti-CCR2 (ab273959) antibody, anti-Ly6G antibody (ab25377), Alexa Fluor® 488-conjugated donkey anti-rabbit antibody (ab150075), SELPLG Antibody (ab2838498), and Alexa Fluor® 647-conjugated donkey anti-mouse antibody were obtained from Abcam (Cambridge, MA, USA). Naringin (Nar, HY-N0153) was obtained from MedChemExpress Co., Ltd. (Shanghai, China). LPS (L2880) was obtained from Sigma (Shanghai, China). 1,2-dipalmitoyl-sn-glycero-3-phosphocholine (DPPC, 850355), Cholesterol (CHO, 228111), and 1,2-dioleoyl-sn-glycero-3-phosphoethanolamine-N-(lissamine rhodamine B sulfonyl) (ammonium salt) (LR-PE, 810150) were obtained from Avanti Polar Lipids (Alabaster, AL, USA). Percoll (D8070-15KU), Wright-Giemsa Stain kit (G1021), Coomassie Brilliant Blue Staining Reagent (P1305), Modified Hematoxylin-Eosin (H&E) staining kit (G1121), BCA protein concentration assay kit (PC0020), and Hoechst 33342 (C0031) were obtained from Solarbio Science & Technology Co., Ltd. (Beijing, China). DCFH-DA (E004-1-1) was from Nanjing Jiancheng Bioengineering Institute (Nanjing, China).

### 2.2 Neutrophil isolation and preparation of neutrophil membrane

Neutrophils were isolated from the bone marrow of healthy male C57BL/6 mice using the percoll gradient method [36]. Firstly, we collected the bone marrow cells from the femurs of the mice, then filtered them through a 70 μm cell strainer, centrifuged, and purified (350×*g*, 5 min, 4°C). Subsequently, the cell suspension was carefully layered on the top of Percoll gradient solution with concentrations of 78%, 68%, and 50%, and centrifuged at 1000×*g* for 30 min at room temperature. The neutrophils were collected on the interface between 78% and 65% layers. After that, neutrophils were washed and removed the red blood cells using RBC lysis buffer. Subsequently, we activated the neutrophils by incubating cells with 200 ng/mL LPS for 2 h. Then, the Wright-Giemsa staining was then used to identify the morphology of neutrophils, and the purified neutrophils were identified by labeling the extracted cells with APC-anti-mouse Ly6G antibody and detecting them using the flow cytometry (AFC2, Invitrogen, Carlsbad, CA, USA).

The activated neutrophil membrane was isolated according to the method described by from Zeng et al. [33]. Briefly, activated neutrophils were suspended in a separation buffer containing 225 mM mannitol, 75 mM sucrose, 0.5 mM EDTA, 30 mM Tris-HCl, and 1 % (v/v) protease inhibitor cocktail. Then, the cell suspension was subjected to ultrasound at 30 W amplitude (2 s on, 3 s off) for 3 min using a probe sonicator on an ice bath. Finally, the suspension was centrifuged at 800×*g* and 10000×*g* for 10 min at 4°C in turns, and the activated neutrophil membrane was collected. At last, The BCA protein concentration assay kit was used to quantify the protein concentration of the cell membrane, and then the activated NM was stored at −80 °C for future use.

### 2.3 Preparation of neutrophil membrane inserted ROS responsive liposome (TK-NLP)

ROS-responsive liposomes (TK-LP) were prepared using the thin-film hydration method [37]. Briefly, 160 μg DPPC, 40 μg DSPE-TK-PEG_2000_, and 40 μg CHO were dissolved in 240 μL dichloromethane-methanol solution (2:1, v/v) to form a lipid film under a nitrogen atmosphere. Subsequently, the lipid film was hydrated with 1 mL of deionized water at 55°C for 30 min and sonicated for 10 min at 55°C in a water bath. After that, we used a liposome extruder (Avanti Lipid, Alabaster, USA) to extrude the mixture of liposome suspension and NM (5: 1 wt%) 20 times through 400 nm and 200 nm polycarbonate membranes in turns, to form TK-NLP.

### 2.4 The characterization of TK-NLP

The morphology of LP, TK-LP, and TK-NLP was observed using transmission electron microscopy (TEM, FEI, TECNAI, G2 F20, USA). Dynamic light scattering (DLS, Malvern Instruments, ZEN 3600, UK) was used to obtain the hydrodynamic diameter and surface charge of these nanoparticles. In addition, to evaluate the ROS-response of TK-NLP, TK-NLP was incubated with 500 μM H_2_O_2_, and then the morphology and size distribution of liposomes were then detected by using TEM and DLS.

### 2.5 Identification of the key membrane proteins in TK-NLP

We used Coomassie brilliant blue staining and western blotting to detect the total proteins and the key membrane proteins including CXCR2, CCR2, LFA-1, PSGL-1, and IL-1R on the TK-NLP. Firstly, neutrophils, NM, and TK-NLP were lysed in RIPA buffer containing protease inhibitor cocktail for 30 min, then mixed with SDS loading buffer and heated at 100°C for 5 min, and the total protein was subjected to SDS-PAGE gel electrophoresis. For total protein staining, the SDS-PAGE gel was stained with Coomassie brilliant blue fast staining solution. For the detection of key membrane proteins, the total protein was transferred to a polyvinylidene difluoride (PVDF) membrane, incubated with antibodies against CXCR2 (1: 1000), CCR2 (1: 1000), IL-1R (1: 500), PSGL-1 (1: 500), and GAPDH at 4°C overnight, respectively. Then, these PVDF were incubated with peroxidase (HRP)-conjugated secondary antibodies at 37°C for 1 h, respectively, and finally, the probed bands were visualized by LI-COR Odyssey imaging system (LI-COR, Nebraska, USA).

### 2.6 In vitro cytokine binding studies

To investigate the nanosponge ability of TK-LP, we mixed 0, 0.5, 1, and 2 mg/mL LP, TK-LP, and TK-NLP with 600 ng/mL TNF-α, IL-1β, and IL-6, respectively. After incubation for 2 h at 37°C, the inflammatory factors adsorbed by the centrifuged nanoparticles were collected. Then the remaining inflammatory factors in the supernatant were detected by the TNF-α, IL-1β, and IL-6 ELISA Kit, respectively.

### 2.7 Encapsulation efficiency and release characteristics

To evaluate the drug loading capacity of TK-NLP, 160 μg DPPC, 40 μg DSPE-TK-PEG_2000_, 40 μg CHO, and 40 μg Nar were dissolved in 280 μL dichloromethane-methanol solution (2: 1, v/v), and the following steps were the same as described in “2.2”. Subsequently, ultrafiltration tubes (Ultracel-®50K) were centrifuged at 7000 rpm at 4°C for 30 min, and the filtrate was collected. Unencapsulated Nar was detected at 263 nm using a microplate reader (TECAN, Groedig, Austria). And the encapsulation efficiency (EE%) of TK-NLP@Nar was calculated with the following equation:

EE% = 100 - Abs263 after centrifuge/Abs263 before centrifuge × 100% (1-1)

To evaluate the drug release characteristics of TK-NLP@Nar, the TK-NLP@Nar was added to a dialysis bag (MW = 3500 DA) and immersed in PBS buffer containing 0, 0.5, or 5 mM H_2_O_2_, the release characteristics of Nar in TK-NLP@Nar were determined at 37°C for 0–12 h. The release characteristics of Nar were further calculated with the following equation:

(1–2) Xn (%) = Cn × V × D/W × 100 %

(1–3) X (%) = Xn+(X1+X2+···Xn-1) × Vi/V

Xn is the degree of released Nar at different time points, Cn is the concentration of Nar measured per time point, V is the volume of the total release medium, D is the dilution factor, W is the initial Nar dosage, X is the cumulative release degree, and Vi is the volume of the release medium took out for per time point.

### 2.8 Hemolysis test

Red blood cells (RBCs) were obtained from SPF healthy male C57BL/6 mice (20–22 g). RBCs were dispersed in normal saline containing sodium heparin and then washed 5 times by centrifugation at 300×*g* for 5 min. After that, 20 μL RBCs suspension was added to 1 mL 10, 100, and 300 μg/mL LP, TK-LP, and TK-NLP normal saline solutions, respectively. 20 μL RBCs suspension was added to normal saline as the negative control, and Triton X-100 solution was added to normal saline as the positive control. The samples were incubated at 37°C for 2 h, and the supernatant was measured at 540 nm after centrifugation at 300×*g* for 5 min. The hemolysis rate was calculated as follows:

(1–4) Hemolysis Rate (%) = (ODs - ODnc) / (ODpc - ODnc) × 100 %

ODs, ODpc, and ODnc represent the absorbance of the sample, positive control, and negative control, respectively.

### 2.9 Cell culture

Human umbilical vein endothelial cells (HUVEC) and human embryonic lung fibroblast cells (MRC-5) were incubated in Dulbecco's Modified Eagle's Medium (DMEM). Human bronchial epithelial cells (16HBE) were cultured in Roswell Park Memorial Institute 1640 medium (RPMI-1640). All cells were cultured in medium supplemented with 10% heat-inactivated fetal bovine serum (FBS), penicillin (100 units/mL), and streptomycin (100 units/mL), at 37°C under humidified conditions of 5% CO_2_ and 95% air.

### 2.10 Cell viability detection

The cytotoxicity of TK-NLP was determined using the CCK-8 assay. 16HBE cells were seeded in a 96-well plate at a density of 1 × 10^4^ cells per well. After 24 h, 0, 12.5, 25, 50, 75, 100, and 250 μg/mL LP, TK-LP, and TK-NLP were incubated with the cells, respectively. After another 24 h, the cells were washed with a warm medium three times and then incubated with 100 μL medium containing 10% (v/v) CCK-8 for 3 h, and the absorbance at 450 nm was measured using a microplate reader.

### 2.11 In vitro targeting of platelets by TK-NLP

Fluorescent lipid LR-PE was prepared by dissolving LR-PE and the mixing lipid (1: 100, mol/mol) mentioned above in a dichloromethane-methanol solution (2: 1, v/v), and the following steps were the same as in part “2.3”. Platelets were obtained from the blood of C57BL/6 mice by collecting whole blood with a solution containing 10–15% ACD (38 mM citric acid, 75 mM sodium citrate, 124 mM glucose) [38]. Platelet-rich plasma was isolated by centrifugation at 200×*g* for 10 min at room temperature, and platelets were resuspended in Buffer A (130.0 mM NaCl, 10 mM citric acid, 9 mM NaHCO_3_, 6 mM glucose, 0.9 mM MgCl_2_, 0.8 mM K_2_HPO_4_, 10 mM (HOCH_2_)_3_CNH_2_). Platelets were incubated with thrombin (0.1 U/mL) for 30 min for platelet adhesion. Unadhered platelets were washed away using Buffer A, followed by co-incubation with LR-PE-labeled LP, TK-LP, TK-NLP + PSGL-1, and TK-NLP for 2 h, respectively. Cells were washed three times with Buffer A and collected. The average fluorescence intensity of each sample was detected using flow cytometry and then quantified using FlowJo V8.

For HCA analysis, incubation was completed as described above. Fixed using 4% paraformaldehyde and treated with Hoechst 33342 for 10 min. The visual images were obtained by the HCA system.

### 2.12 Effect of TK-NLP on platelet-neutrophil interactions *in vitro*

To assess the effect of nanoparticles on platelet-neutrophil complex formation, platelets and neutrophils were collected according to the same method as in “2.11” and “2.2”. And the platelets were labeled using Dio, LP, TK-LP, TK-NLP + PSGL-1, and TK-NLP were co-cultured with neutrophils and platelets at 37°C for 2 h, respectively. Subsequently, LPS (100 ng/mL) and thrombin (0.1 U/mL) were added to the culture medium for activation of neutrophils and platelets. At the end of the incubation, neutrophils were fixed using 4% paraformaldehyde and treated with Hoechst 33342 for 10 min to label the nucleus of neutrophils. Visual images were obtained using the HCA system.

For the flow cytometry assay. Neutrophils were labeled with Dil, and platelets were labeled with Dio. LP, TK-LP, TK-NLP + PSGL-1 and TK-NLP were co-cultured with neutrophils and platelets for 2 h at 37°C, respectively. Subsequently, LPS (100 ng/mL) and thrombin (0.1 U/mL) were added for activation. At the end of the incubation, the cells were washed and collected. The average fluorescence intensity of each sample was detected using flow cytometry and quantified using FlowJo V8.

### 2.13 Establishment of LPS-induced 16HBE model

16HBE were seeded into a 96-well plate at a density of 1 × 10^4^ cells per well. After incubation for 12 h, cells were incubated with 10 μg/mL LPS for 24 h. And then, cells were incubated with 10 μM DCFH-DA for 30 min. After that, the visual images were obtained by the Operetta High Content Analysis (HCA) system (PerkinElmer, Boston, MA, USA).

### 2.14 Preparation of mimicking lung organ (mLO)

We prepared the dopamine-modified sodium alginate (P-Alg) microsphere cell scaffold using an in-house protocol [39,40]. Firstly, anionic polymer sodium alginate (Alg) microspheres were prepared under the following conditions using a 3D bioprinter (RegenHU SA, Z.l. du Vivier 22, CH-1690 Villaz St Pierre, Switzerland). Subsequently, type I collagen was added to a centrifuge tube containing Alg microspheres, vortexed vigorously, and settled under gravity at 4°C. After that, excess type I collagen was removed by centrifugation at 4°C and microspheres were incubated with a dopamine solution for 2 h. Finally, the re-prepared P-Alg microspheres were washed three times, dispersed in a cell culture medium, and stored at 4°C.

Subsequently, we prepared mLO by mixing cells of 16HBE, HUVEC, and MRC5 in a ratio of 25: 25: 50. Then, 1 × 10^5^ mixed cells and approximately 50 P-Alg microspheres were combined and cultured in a 96-well U-shaped plate for 24 h.

### 2.15 Targeting of TK-NLP to 16HBE and mLO models *in vitro*

16HBE cells and mLO were incubated with LPS for 12 h. Subsequently, LR-PE labeled LP, TK-LP, TK-NLP + PSGL-1 and TK-NLP were incubated with the cells for 1, 2, and 4 h, respectively. Then, 16HBE cells and mLO were fixed with 4% paraformaldehyde after being washed with a warm medium three times, and treated with Hoechst 33342 for 10 min. The visual images were obtained by the HCA system and quantified using ImageJ software.

For flow cytometry assay, following the same procedure. After incubation, mLO and 16HEB cell were collected and digested using trypsin at 37°C for 5 min. We used flow cytometry to detect the average fluorescence intensity of each cell and then quantified using FlowJo V8.

### 2.16 In microfluidic organ chip system targeting of TK-NLP to mLO

The microfluidic organ chip system was used in our previous study [40]. Nanoparticles were dispersed in the culture medium and passed over the organ chip at a flow rate of 0.4 mL/min for 2 h using a microfluidic system. After that, the mLO was transferred into the organ chip and incubated with nanoparticles in the chip with 0.4 mL/min medium flow for 1, 2, and 4 h, respectively. Subsequently, the mLO were digested using trypsin at 37°C for 5 min to obtain a single-cell suspension. The cells were washed with PBS buffer twice, and the fluorescence intensity was detected by flow cytometry and analyzed by Flowjo V10.

### 2.17 ROS and neutrophil detection in lung tissues

The mouse lung tissue was cut and ground, filtered through a Corning®70 μm cell strainer, and then centrifuged to collect the cells. After that, the cells were incubated with 10 μM DCFH-DA at 37°C for 30 min and then washed with PBS buffer. The fluorescence intensity was detected by flow cytometry and analyzed by Flowjo V10 software.

The pre-treatment steps for lung tissue were the same as “2.17” to obtain lung cells, and then were incubated with APC-anti-mouse Ly6G Antibody (1: 200) on ice for 30 min, and then washed with PBS buffer. The fluorescence intensity was detected by flow cytometry and analyzed using FlowJo V10 software.

### 2.18 In vivo distribution of TK-NLP

We established the ALI model in mice, and the LR-PE-labeled TK-NLP, TK-LP, and LP were intravenously injected into the ALI mice. Then, the fluorescent images were acquired using an *in vivo* imaging system (IVIS, Lumina K Series III, PerkinElmer) at 3, 6, 12, and 24 h. After 24 h, the mice were euthanized, and the heart, liver, spleen, lung, and kidney were collected for fluorescence intensity observation using IVIS. Finally, lung tissue sections were obtained using a freezing microtome (Microm HM 550, USA). The distribution of TK-NLP in the lung tissue sections was observed by an inverted fluorescence microscope (Leica Microsystems CMS GmbH, ErnstLeitz-Str. 17–37, Germany).

### 2.19 Animal experiments

C57BL/6J mice (male, 18–20 g) were purchased from SPF Biotechnology Co., Ltd. (Beijing, China). The mice were kept at Tianjin University of Traditional Chinese Medicine Animal Center, conducting experiments in the rearing environment of the breeding room and the ethical system stipulated by the Animal Ethics Committee. 4 mg/kg LPS was administered via tracheal drip to establish the ALI model. After 12 h, the mice were intravenously injected with 300 μL PBS buffer (control group), Nar, LP@Nar, TK-LP@Nar, NLP@Nar, and TK-NLP@Nar with a dose of 8 mg/kg Nar, respectively. After 48 h of LPS treatment, micro-CT (QuantumFX-CT Software, PerkinElmer, USA) was performed to observe the lungs of mice, then euthanize and collect blood, followed by obtaining bronchoalveolar lavage fluid (BALF). All experiments were approved by the Institutional Animal Care and Use Committee and were conducted according to the guidelines of Tianjin University of Traditional Chinese Medicine.

### 2.20 Total cells and protein concentrations in BALF

The collected BALF was centrifuged at 350×*g* for 5 min at 4°C, the supernatant was collected, and the protein content in the supernatant was detected using the BCA protein assay kit. The cells were resuspended in PBS buffer and the total cell number was detected by flow cytometry.

### 2.21 Lung wet/dry (W/D) ratio

The lung W/D ratio was assessed to reflect the severity of pulmonary edema. The right lung middle lobe was weighed as wet weight. The lung tissues were then dried at 60°C for 48 h to obtain the dry weight. The W/D ratio was calculated as wet weight/dry weight of lung tissues.

### 2.22 Hematoxylin-Eosin (H&E) staining

The lung tissue sections were stained using the modified H&E staining kit. The lung tissue sections were treated with hematoxylin staining for 60 s, 1% hydrochloric acid ethanol (v/v) solution for 3 s, cyanation solution for 3 s, and eosin staining for 120 s in turn. After each step, lung tissue sections were washed with running water for 30 s. Finally, lung tissue sections were observed using an inverted fluorescence microscope.

### 2.23 Immunofluorescence (IF) staining

For IF staining, non-specific antibody binding was blocked by incubating the sample with 10% FBS for 1.5 h at room temperature. After that, lung tissue sections were incubated with ZO-1 (1: 200), E-cadherin (1: 200), and Ly6G primary antibodies (1: 200) at 4°C overnight, respectively. Subsequently, lung tissue sections were incubated with Alexa Fluor® 488-conjugated goat anti-rabbit secondary antibody (1: 200) or/and Alexa Fluor®594-conjugated goat anti-rat secondary antibody (1: 200) for 60 min at 37°C, respectively, and nuclei were stained with Hoechst 33342. Finally, the lung tissue sections were observed using an upright fluorescence microscope (Leica THUNDER, Germany) and quantitative analysis using ImageJ software.

### 2.24 Enzyme-linked immunosorbent assay (ELISA)

ELISA kit was used to detect the concentrations of TNF-α, IL-1β, and IL-6 in serum and BALF. The collected blood and BLAF were centrifuged at 1000×*g* for 10 min at 4°C, the supernatant was collected, and then followed by incubation with capture antibody, detection antibody, avidin HRP, TMB substrate, and termination solution were added according to the instructions of the kit. Finally, the absorbance at 450 nm was measured using a microplate reader.

### 2.25 Quantitative real-time polymerase chain reaction (RT-PCR)

20 mg of lung tissue was sheared, and total RNA was extracted from lung tissue using TRIzol® reagent (15596018cn, Invitrogen). RNA samples (1 μg) were then reverse transcribed using the Transcriptor first strand cDNA synthesis Kit (Roche, Mannheim, Germany) synthesis kit. The obtained cDNA was incubated with Bestar® SYBR Green qPCR Master Mix(DBI®Bioscience, Ludwigshafen, Germany) and placed in LightCycler 480 II (Roche, Mannheim, Germany) for amplification. Data were analyzed using the comparative CT method (2^−ΔΔCT^). Primer sequences are listed in Table S1.

### 2.26 In vivo safety

Healthy mice were intravenously injected with PBS buffer (control group), and TK-NLP. After 14 days, the whole blood from the mice was collected for complete blood count analysis. The main indicators including blood cell analysis (white blood cells (WBC), red blood cells (RBC), hemoglobin (HGB), platelets (PLT). The main indicators include aspartate aminotransferase (AST), alanine aminotransferase (ALT), blood urea nitrogen (BUN), creatinine (CRE), and creatine kinase (CK). Finally, the main organs of mice were collected, and the organ index was calculated according to the organ weight according to the following formula:

Organ index (%) = organ weight × 100 %/ body weight (1–5)

### 2.27 Statistical analysis

All data were expressed as the mean ± SD from at least three independent experiments. Comparisons between two groups were performed using a *t*-test, with *P* < 0.05 considered statistically significant. For comparisons involving more than two groups, use one-way analysis of variance (ANOVA), followed by Tukey's test for multiple comparisons. All analyses were conducted using GraphPad Prism 9.5 (GraphPad Software, San Diego, CA, USA).

## 3 Results and discussion

### 3.1 Preparation and characterizations of TK-NLP

**Fig. 1:**
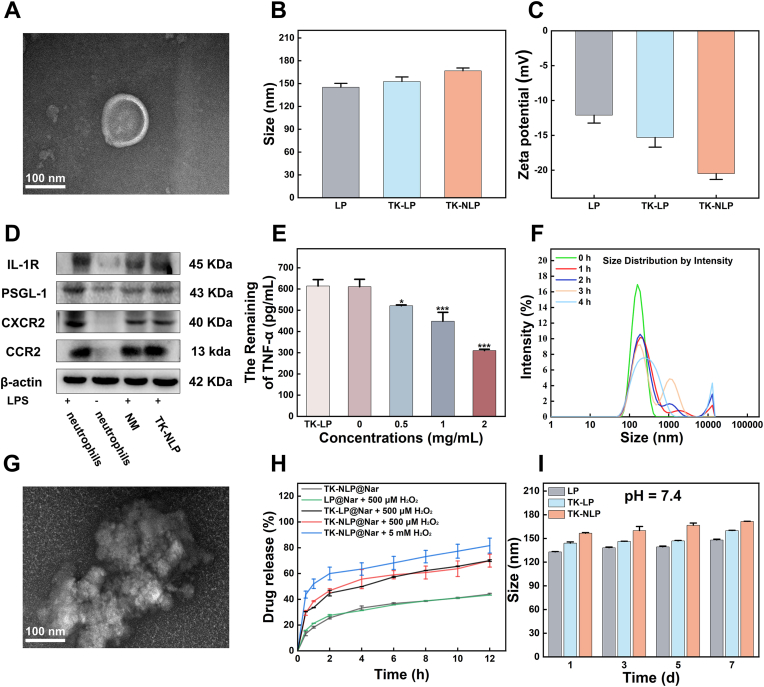
Preparation and characterizations of TK-NLP. (**A**) TEM image of TK-NLP. (**B**) The size and (**C**) surface charge of LP, TK-LP, and TK-NLP were detected by DLS. (**D**) The detections of membrane receptor proteins including CCR2, IL-1R, PSGL-1, and CXCR2 in neutrophils (LPS+), neutrophils (LPS-), NM (LPS+), and TK-NLP (LPS+). (**E**) After co-cultured with 2 mg/mL TK-LP and different concentrations of TK-NLP, the remained concentrations of TNF-α were detected by ELISA. (**F**) The particle size distributions of TK-NLP after incubation with 1 mM H_2_O_2_ for 0, 1, 2, 3, and 4 h. (**G**) TEM image of TK-NLP after incubated with 500 μM H_2_O_2_. (**H**) The release curves of LP@Nar, TK-LP@Nar, and TK-NLP@Nar with varying concentrations of H_2_O_2_. (**I**) The size changed of LP, TK-LP, and TK-NLP in 7 days were detected using DLS at pH = 7.4. Values shown are mean ± S.D., The experiments were performed in three replicates. Scare bar = 100 nm.

During inflammation, neutrophils receive signals to respond rapidly and infiltrate into inflammatory tissues in large numbers, and relevant studies have shown that activated neutrophils can increase the targeting of inflammation sites [33]. Neutrophils were isolated from mouse bone marrow using the Percoll density gradient neutrophil isolation method and the flow cytometry analysis showed high purity of neutrophils (Fig. S1). After activation with LPS, the neutrophils developed pseudopodia, indicating neutrophil activation (Fig. S2). After that, we isolated the activated neutrophils membrane and prepared the neutrophil membrane-camouflaged ROS-responsive liposome (TK-NLP) that showed characteristic spherical structure (Fig. 1A). DLS detected the size of TK-NLP that was enhanced to 166.71 ± 3.77 nm after being fusion with the neutrophil membrane compared with TK-LP (152.71 ± 6.01 nm) (Fig. 1B). Also, the surface charge of LP (−12.10 ± 1.13 mV) was decreased into −15.30 ± 1.39 mV (TK-LP) after incorporating the ROS-responsed lipid, and further decreased into −20.49 ± 0.84 mV (TK-NLP) after camouflaged with neutrophil membrane with negative surface charge (Fig. 1C). Also, the flow cytometry was employed to verify the successful fusion of neutrophil membrane, compared to TK-LP and showed more than 90% fusion rate (Fig. S3). Importantly, TK-NLP showed a similar pattern of protein bands with the neutrophil membrane, indicating TK-NLP inherited most of the membrane proteins (Fig. S4). And some key membrane receptor proteins such as IL-1R, CCR2, PSGL-1, and CXCR2 were shown on TK-NLP, and these protein receptors were significantly upregulated after LPS stimulation (Fig. 1D–S5). Therefore, TK-NLP could act as the nanosponge to absorb the inflammatory cytokines such as TNF-α, IL-6 and IL-1β indicating these retained protein receptors maintained their functionality (Fig. 1E and S6). These results confirmed the successful preparation of TK-NLP with retained integrity and functionality of neutrophil membrane receptor proteins.

After that, we examined the ROS responsiveness of TK-NLP by detecting changes in the morphology and size distribution in high ROS environment. Results showed that the particle size distribution of TK-NLP changed significantly within the first 1 h (Fig. 1F), and the morphological changes were also observed in the TEM image (Fig. 1G). Additionally, the drug loading of TK-NLP@Nar was measured as 9.45 ± 1.11%, with an encapsulation efficiency (EE%) of 22.98 ± 2.99%. The release rates of TK-NLP@Nar were 70.01 ± 4.99% and 81.71 ± 5.80% under 500 μM and 5 mM H_2_O_2_ after 12 h, respectively, which were significantly reduced in these controls without ROS or ROS-responsive lipid to 43.95 ± 0.68% and 43.30 ± 0.15%, respectively. Moreover, the TK-NLP@Nar (70.01 ± 4.99%) and TK-LP@Nar (70.03 ± 0.67%) showed similar release rates in 500 μM H_2_O_2_ environment for 48 h, indicating that the fused neutrophil membrane showed no effect on the ROS response ability (Fig. 1H). These results demonstrated that the TK-NLP@Nar showed ROS-responsive drug release ability. After that, we assessed the hemocompatibility and stability of TK-NLP. The hemolysis rates of LP, TK-LP, and TK-NLP were all below 5%, showing minimal hemolytic risk (Fig. S7). DLS measurements revealed that the particle size of TK-NLP remained at around 150 nm over a 7-days period and in different pH environments. The minor size changes observed indicated good stability (Fig. 1I–S8). In summary, the TK-NLP nanoparticles we developed retained integral neutrophil receptor proteins, excellent ROS responsiveness, high stability, and hemocompatibility.

### 3.2 Effect of nanoparticles on PNAs

**Fig. 2:**
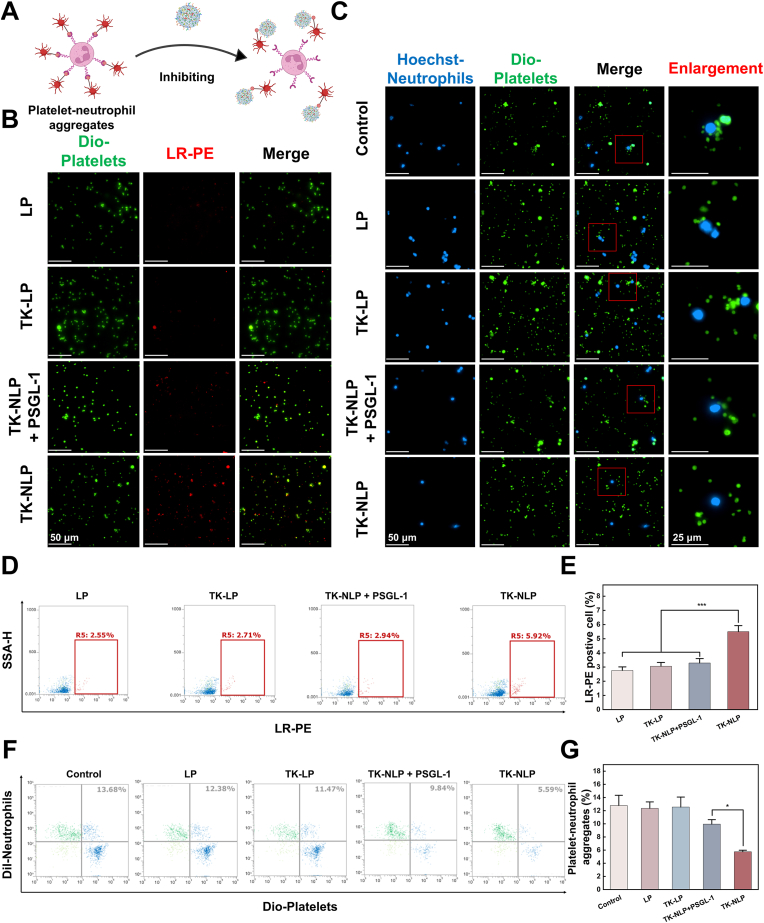
Effect of TK-NLP on PNAs in vitro. (**A**) Schematic illustration of TK-NLP competitive binding to platelets, inhibiting PNAs formation. (**B**) Fluorescence images of Dio-labeled platelets after co-incubation with LR-PE labeled LP, TK-LP, TK-NLP + PSGL-1, and TK-NLP, respectively. (**C**) Fluorescence images of Hoechst-labeled neutrophils and Dio-labeled platelets incubated with LP, TK-LP, and TK-NLP, respectively. (**D**) Flow cytometry analysis of Dio-labeled platelets after co-incubation with LR-PE labeled LP, TK-LP, TK-NLP + PSGL-1, and TK-NLP, respectively, and the (**E**) quantitative analysis results (n = 3). (**F**) Flow cytometry analysis of Dil-labeled neutrophils and Dio-labeled platelets incubated with LP, TK-LP, TK-NLP + PSGL-1, and TK-NLP, respectively, and the (**G**) quantitative analysis results of the formation of PNAs (n = 3). Values shown are mean ± S.D., ∗*P* < 0.05, ∗∗∗*P* < 0.001. Scale bar = 50 μm/25 μm.

PNAs are often required for cell recruitment to the site of inflammation, and platelet activation is increased in patients with ALI [41]. Neutrophil nanoparticles can efficiently inherit proteins from activated neutrophils, suggesting that nanoparticles may have a targeting ability to platelets and reduce the formation of neutrophil-platelet aggregates by competing with neutrophils to bind platelets (Fig. 2A). We compared the binding ability of TK-NLP with TK-LP and LP to platelets *in vitro*, and incubated Dio-labeled platelets with LR-PE-labeled nanoparticles. Results showed that the highest red fluorescence signal was observed on platelets in the TK-NLP group, indicating that TK-NLP could significantly enhance binding ability to platelets compared with TK-LP and LP (Fig. 2B). To determine whether neutrophils bind to platelets cells via the PSGL-1 integrin, we first pretreated TK-NLP with anti-PSGL-1 antibody before co-culturing with platelets. The anti-PSGL-1 antibody significantly reduced the fluorescence intensity of TK-NLP in the co-culture system, indicating impaired binding capacity to platelets (Fig. 2B). Flow cytometry further confirmed that the binding ability of TK-NLP for platelets was much higher than that of the TK-LP group (5.51 ± 0.42% vs. 2.76 ± 0.24%, *P* < 0.001) and the LP group (5.51 ± 0.42% vs. 3.05 ± 0.27%, *P* < 0.001) (Fig. 2D and E).

To investigate whether TK-NLP possesses the ability to inhibit PNA formation, we conducted a competitive binding experiment with platelets to inhibit the formation of PNAs. Results showed that Dil-labeled platelets could adhere to neutrophils in control group, and the number of platelets adhering to neutrophils was reduced by the addition of TK-NLP. After incubation with TK-NLP and PSGL-1 antibodies, platelets re-adhere to neutrophils (Fig. 2B). Quantitative results obtained by flow cytometry showed that TK-NLP could effectively reduce the formation of PNAs compared to the TK-LP group (7.52 ± 2.05% vs. 11.98 ± 1.56%, *P* < 0.001) and the LP group (7.52 ± 2.05% vs. 11.8 ± 1.67%, *P* < 0.001) (Fig. 2F and G), indicating that TK-NLP could reduce PNAs production.

### 3.3 Targeting and therapeutic efficacy of TK-NLP@Nar in 16HBE

To study the molecular mechanisms and pathological process of LPS-induced ALI, human bronchial epithelial cell line 16HBE is commonly used as an *in vitro* model [42,43]. Consequently, we chose to utilize LPS-induced 16HBE cell models for subsequent experiments. The cell survival rates were continuously decreased with the increased concentrations of LP and TK-LP. In contrast, the addition of neutrophil cell membranes was found to be significantly reduced in the TK-NLP group, thereby mitigating its potential for toxicity (Fig. S9). In addition, the ROS levels of 16HBE cells were significantly elevated after stimulation with LPS (Fig. S10). This indicated that we have successfully constructed an *in vitro* inflammation model that can produce high ROS levels.

**Fig. 3:**
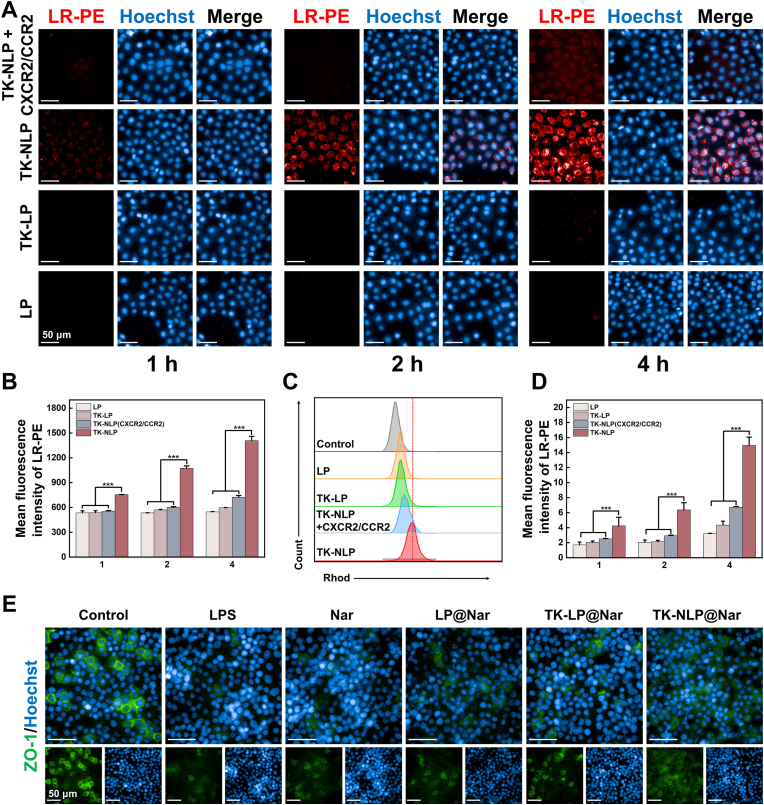
Targeting and therapeutic efficacy of TK-NLP@Nar *in vitro*. (**A**) Images of LPS-induced 16HBE cells after incubated with LR-PE labeled LP, TK-LP, and TK-NLP for 1, 2, and 4 h, and (**B**) quantification of the mean fluorescence intensity of LR-PE (n = 5). (**C**) Flow cytometry analysis of 16HBE cells after incubated with LR-PE labeled LP, TK-LP, and TK-NLP for 4 h, and (**D**) quantification of fluorescence intensity of LR-PE at 1, 2, and 4 h (n = 5).(E) Immunofluorescence staining images of ZO-1 protein. Values shown are mean ± S.D., ∗∗∗*P* < 0.001. (For interpretation of the references to color in this figure legend, the reader is referred to the Web version of this article.)

Subsequently, we evaluated the targeting ability of TK-NLP using the LPS-induced injury model based on 16HBE cells. Fluorescent lipid LR-PE labeled LP, TK-LP, and TK-NLP nanoparticles were incubated with the injury model for 1, 2, and 4 h, respectively. The results showed that 16HBE cells exhibited slow uptake of LP and TK-LP nanoparticles. In contrast, the fluorescence intensity of 16HBE cells was continuously increased with time in the TK-NLP group and was significantly higher than the LP and TK-LP groups at each time point (Fig. 3A and B). We further validated the targeting efficiency of TK-LP using flow cytometry (Fig. 3C and D). To determine whether neutrophils target 16HBE cells for binding by receiving inflammatory signals such as CXCR2/CCR2, we pre-treated TK-NLP cells with anti-CXCR2/CCR2 antibodies before co-culturing them with 16HBE cells. The anti-CXCR2/CCR2 antibody significantly reduced the fluorescence intensity of TK-NLP cells in the co-culture system, indicating impaired ability to target 16HBE cells (Fig. 3A and B). Consistent with the above results, the fluorescence intensity in the TK-NLP group showed 1.56-, 2.05-, and 2.35-folds higher than the TK-LP group in 1, 2, and 4 h, respectively. Also, we found the fluorescence intensity of cells in the TK-LP group was significantly higher than the LP group at 4 h, likely the release of LR-PE after responding to the ROS expressed by 16HBE cells (Fig. 3D). Therefore, more Nar can be delivered into LPS-induced 16HBE cells using the TK-NLP nanoparticles, promoting ZO-1 expression to repair the lung barrier (Fig. 3E and S11).

### 3.4 Targeting and therapeutic efficacy of TK-NLP@Nar in mLO

**Fig. 4:**
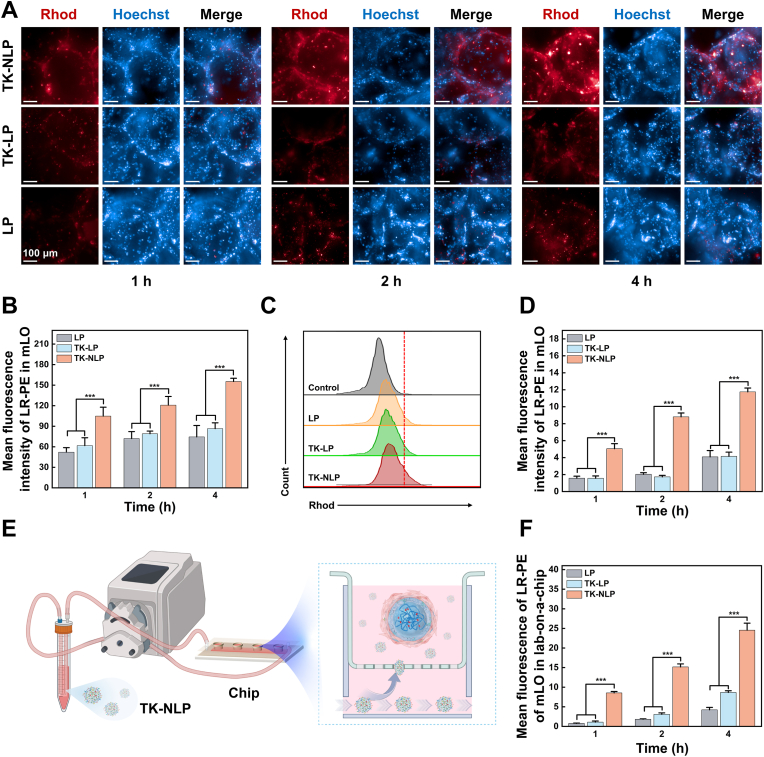
Targeting efficacy of TK-NLP on a 3D ALI model based on mLO and in a chip with a dynamic cultivation system *in vitro*. (**A**) Images of mLO after incubation with LR-PE labeled LP, TK-LP, and TK-NLP for 1, 2, and 4 h, and (**B**) quantification of the mean fluorescence intensity of LR-PE (n = 5). (**C**) Flow cytometry analysis of mLO after incubation with LR-PE labeled LP, TK-LP, and TK-NLP for 4 h, and (**D**) quantification of fluorescence intensity of LR-PE at 1, 2, and 4 h (n = 5). (**E**) Schematic diagram of nanoparticle uptake by mLO in a chip with a dynamic cultivation system. (**F**) Quantitative analysis of the mean fluorescence of LR-PE of mLO in a chip using flow cytometry after incubation with LR-PE labeled LP, TK-LP, and TK-NLP for 1, 2, and 4 h (n = 5). Values shown are mean ± S.D., ∗∗∗*P* < 0.001. Scare bar = 100 μm.

Traditional drug efficacy assessment and formulation evaluation methods typically rely on 2D cellular and animal models, and existing shortcomings make it difficult to meet clinical requirements for screening data accuracy [44,45]. Organoids, an essential bridge between 2D cellular and animal models, are more physiologically relevant than 2D culture models and easier than animal models for manipulating ecological niche composition, signaling pathways, genome editing, etc. [46,47]. In this study, we previously developed a mimicking lung organ (mLO) based on 3D-printed hydrogel microspheres in our group, which we used to establish an ALI model *in vitro*. We used the ALI model to evaluate the targeting efficiency of TK-NLP *in vitro*. LR-PE labeled LP, TK-LP, and TK-NLP were incubated with the mLO for 1, 2, and 4 h, respectively, and results showed that the uptake of TK-NLP by mLO was significantly higher than that of LP and TK-LP at each time point (Fig. 4A and B). The fluorescence intensity of mLO in the TK-NLP group was 1.49-, 1.38-, and 1.45-folds higher than that in the TK-LP group in 1, 2, and 4 h, respectively, after the quantitatively analyzed using flow cytometry (Fig. 4C and D). These results indicated that the neutrophil membrane endowed TK-NLP with superior targeting ability and strong targeting specificity within the simulated lung environment. Furthermore, TK-NLP@Nar notably increased the expression of tight junction proteins E-Cadherin and ZO-1 in mLO, effectively restoring the barrier function of the mimicking lung organ (Fig. S12).

Subsequently, a microfluidic system was employed to mimic blood circulation, with mLO positioned within the chip to emulate lung organs. This configuration provide a more authentic simulation of the pulmonary environment, enabling the evaluation of the targeting of TK-NLP to lung tissues *in vivo*. The mLO was placed in the upper chamber and then released chemokines after the stimulation of LPS, while the culture medium flowed in the lower chamber, and TK-NLP migrated and targeted the mLO through these chemokines after the TK-NLP nanoparticles were added into the culture medium (Fig. 4E). In a similar experiment, LR-PE labeled LP, TK-LP, and TK-NLP were added to the culture medium, and the fluorescence intensity of LR-PE in mLO was then measured at 1, 2, and 4 h using flow cytometry. The results demonstrated that the fluorescence intensity in mLO of the TK-NLP group also exhibited 2.32-, 2.81-, and 1.62-folds increases compared to the TK-LP group at 1, 2, and 4 h, respectively, in the dynamic environment (Fig. 4F). In summary, the TK-NLP demonstrated a novel targeting capability for injured lung tissues.

### 3.5 In vivo distribution of nanoparticles

**Fig. 5:**
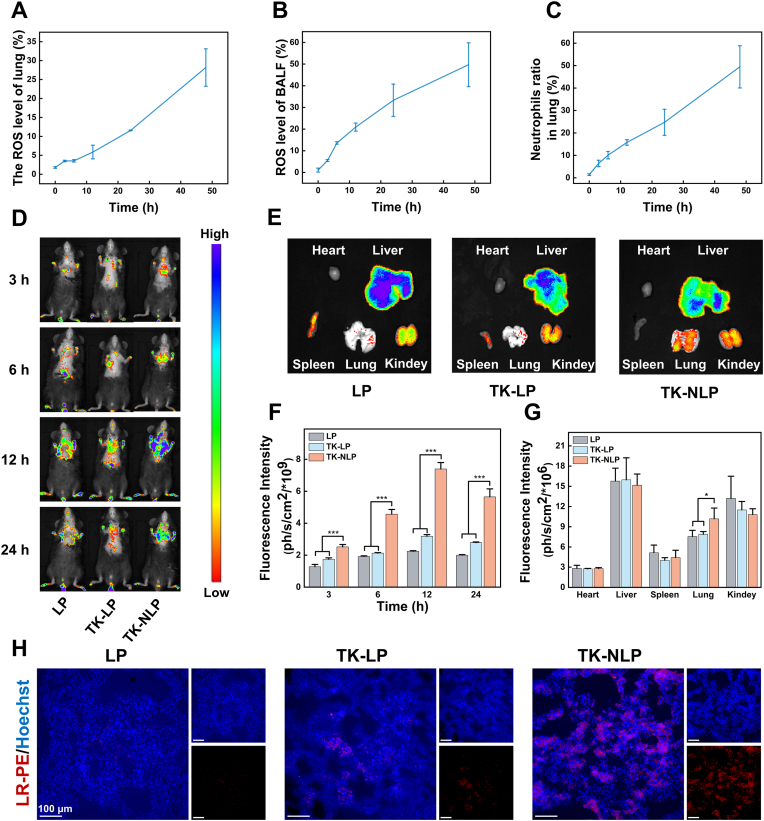
Targeting injured lung sites of TK-NLP i*n vivo*. (**A**) Measurement of ROS content in lung and (**B**) in BALF at 0, 3, 6, 12, 24 and 48 h of LPS-induced ALI mice (n = 4). (**C**) Neutrophils ratio in the lung tissue at 0, 3, 6, 12, 24 and 48 h of LPS-induced ALI mice (n = 4). (**D**) The fluorescence images were detected by IVIS at 3, 6, 12, and 24 h of LPS-induced ALI mice. (**E**) The fluorescence images of the heart, liver, spleen, lung, and kidney were isolated from mice at 24 h. (**F**) The quantification of fluorescence intensity in the lung site at different time-points *in vivo* (n = 4) and (**G**) the isolated organs *in ex-vivo* (n = 4). (**H**) The fluorescence images of lung sections after being injected with these nanoparticles at 24 h. Values shown are mean ± S.D., ∗*P* < 0.05, ∗∗∗*P* < 0.001. Scare bar = 100 μm.

Next, we evaluated the targeting ability of TK-NLP in the LPS-induced ALI mouse model *in vivo*. Before that, we confirmed whether more neutrophils were migrated and more ROS produced in the lung tissue of the ALI mouse model we prepared. We administered LPS *via* tracheal instillation to mice, and we observed a sustained and significant increase in ROS levels in lung tissue and BALF in 0–48 h (Fig. 5A and B). Additionally, the ratios of neutrophils in lung tissues increased continuously from 0-48 h (Fig. 5C). These results indicated the migration and infiltration of neutrophils in injured lung tissues, as well as the significant production of ROS in lung tissue in the ALI model we constructed.

LR-PE labeled LP, TK-LP, and TK-NLP were intravenously injected into LPS-induced ALI mice, and the targeting efficiency of these nanoparticles was studied by monitoring the fluorescence distribution using IVIS at 3, 6, 12, and 24 h. As shown in Fig. 5D and F, both TK-LP and TK-NLP reached peak accumulation in the lung at 12 h, followed by a gradual decline, and TK-NLP showed the highest accumulation in the lung tissue at all time points compared with other groups. In addition, we found that TK-NLP accumulated more in the lungs than LP at 12 and 24 h, due to TK-NLP responded to the ROS environment, which triggered the release and accumulation of LR-PE at the lung sites. Fluorescence imaging and quantification of main organs at 24 h revealed that TK-NLP accumulated significantly more in the lungs than in other groups (Fig. 5E–G). The modified neutrophil cell membrane significantly improved the retention time of nanoparticles in the lung compared with the TK-LP group, which can fully meet the time window for nanoparticles to trigger ROS. Subsequently, in lung tissue frozen sections and fluorescence analysis, the TK-NLP group showed a more obvioused fluorescence signal than the other two groups, which was consistent with IVIS imaging results, further confirming the targeting ability of the drug delivery system to the inflammatory lung (Fig. 5H).

### 3.6 Therapeutic effects of TK-NLP on ALI

**Fig. 6:**
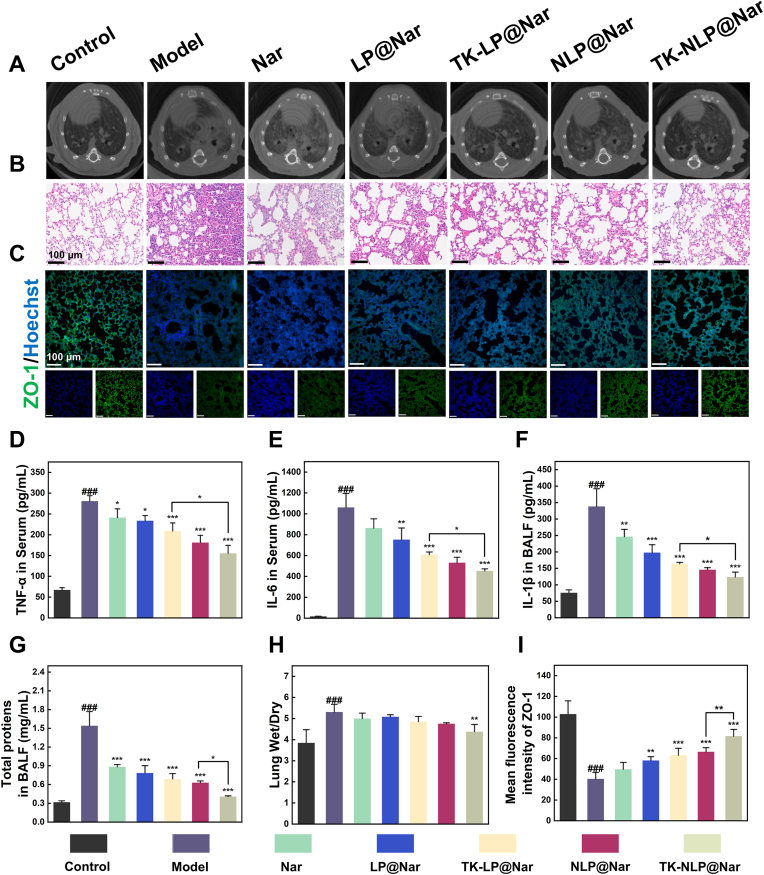
Therapeutic effects of TK-NLP@Nar on LPS-induced ALI mice. (**A**) Micro-CT images and (**B**) H&E staining images of lung tissue. (**C**) The immunofluorescence images of ZO-1 in lung tissue. ELISA assay measured the concentrations of (**D**) TNF-α and (**E**) IL-6 in serum, and (**F**) IL-1β in BALF (n = 4). (**G**) Total proteins in BALF (n = 4). (**H**) Lung W/D ratio (n = 4). (I) Qualification analysis of mean fluorescence intensity of ZO-1 (n = 4). Values shown are mean ± S.D., ^###^*P* < 0.001, vs Control group, ∗*P* < 0.05, ∗∗*P* < 0.01, ∗∗∗*P* < 0.001, vs Model group. Scale bar = 100 μm.

After validated the lung-targeting and ROS-response properties of TK-NLP, we evaluated the therapeutic efficacy of this Nar delivery system on the ALI mouse model *in vivo*. We injected free Nar or nanoparticles with 8 mg/kg Nar *via* the tail vein of mice after LPS tracheal instillation for 4 h. After 48 h, we observed the structural changes of lung tissue using micro-CT and H&E staining. Micro-CT results revealed diffuse infiltration and increased density shadows of the mice lungs after treated mice with LPS, and the lung shadows exhibited a reduction in the TK-NLP@Nar group (Fig. 6A). After that, we observed the histopathological changes of lung tissue were examined *via* H&E staining. The lung tissue structure in the model group collapsed and significantly increased in lung tissue area compared to the control group. In contrast, the TK-NLP@Nar group exhibited a reduction in lung tissue structure damage and interstitial hyperplasia (Fig. 6B).

Furthermore, after being treated with LPS, the total numbers of cells in the BLAF significantly increased to approximately 6.62-folds than that of the control group. After administration, the total numbers of cells in BALF were all decreased, with the TK-NLP@Nar group showing significantly reduction than the model group (0.71 ± 0.04 vs 1.99 ± 0.99, *P* < 0.001) (Fig. S13). In addition, in the lung tissue of LPS-induced ALI mice, a significant increase in the number of neutrophils was observed than the control group (32.22 ± 6.78 vs 3.64 ± 1.05, *P* < 0.001). Then, we observed the significant decrease in neutrophil infiltration in lung tissues of the TK-NLP@Nar group than the model group (11.67 ± 1.86 vs 32.22 ± 6.78, *P* < 0.001) and NLP@Nar group (11.67 ± 1.86 vs 15.91 ± 1.23, *P* < 0.01), indicated that TK-NLP@Nar could effectively inhibit neutrophil infiltration in lung tissues of ALI mouse model (Fig. S14).

Subsequently, we detected the concentrations of inflammatory factors in mouse BALF and serum (Fig. 6D–F, S15, S16). Results showed that the inflammatory factors IL-6, TNF-α, and IL-1β were increased by 4.17-, 64.36-, and 1.81-folds in serum, 17.3- and 4.46-folds in BALF of mice after LPS tracheal instillation, respectively. The Nar was able to significantly reduce the expression of inflammatory factors both in BALF and serum after delivered by these DDS. In particular, the TK-NLP@Nar group significantly decreased the section of IL-6 (310.57 ± 38.24 vs 561.77 ± 36.32, *P* < 0.001), TNF-α (452.47 ± 20.15 vs 1061.56 ± 133.98, *P* < 0.001), and IL-1β (23.54 ± 2.51 vs 20.90 ± 1.93, *P* < 0.001) in serum, TNF-α (180.44 ± 53.70 vs 411.97 ± 20.19, *P* < 0.001) and IL-1β (123.57 ± 13.98 vs 338.42 ± 53.98, *P* < 0.001) in the BALF, respectively, which was lower than other DDS groups in IL-1β, IL-6, and TNF-α. Furthermore, TK-NLP@Nar also significantly reduced and inhibited the expression of inflammatory cytokine genes in lung tissue, and the inhibition efficiency was higher than other treatment groups (Fig. S17). These results collectively suggested that TK-NLP@Nar significantly reduced the expression of inflammatory factors in the mouse model of ALI.

The characteristic of ALI is the increased permeability of the capillary endothelium-alveolar epithelium barrier, leading to the influx of protein-rich edema fluid into the air spaces, thereby causing pulmonary edema [48]. Next, we analyzed modified μCT images to observe the leakage areas in the lungs of mice and applied pseudo-coloring (green) to indirectly assess the severity of pulmonary edema. Compared to the model group and other treatment groups, the TK-NLP@Nar group more effectively reduced the leakage area in the lungs (Fig. S18). We analyzed the total protein concentrations in BALF, that in the model group was significantly higher than control group (1.54 ± 0.23 vs 0.32 ± 0.02, *P* < 0.001), and TK-NLP@Nar significantly reduced the total protein concentrations (0.41 ± 0.01 vs 1.54 ± 0.23, *P* < 0.001) in BALF (Fig. 6G). After that, we measured the lung W/D ratio, a typical indicators of pulmonary edema. After LPS administration, the lung W/D ratio was significantly improved in the model group (5.31 ± 0.36 vs 3.85 ± 0.63, *P* < 0.001). In contrast, the TK-NLP@Nar group effectively reduced the W/D ratio (4.38 ± 0.34 vs 5.31 ± 0.36, *P* < 0.01) (Fig. 6H). To further explore the impact of TK-NLP@Nar on lung barrier protection and repair, we analyzed the expression of the tight junction protein ZO-1 in mouse lung tissue using immunofluorescence staining. The results showed that the expression of ZO-1 in the model group was significantly reduced after LPS tracheal insillaion (40.39 ± 6.23 vs 102.91 ± 12.79, *P* < 0.001), means the destruction of the lung barrier. The TK-NLP@Nar group showed 1.57-folds increased expression of ZO-1 than the model group (81.68 ± 6.43 vs 40.39 ± 6.23, *P* < 0.01). (Fig. 6C–I). Furthermore, TK-NLP@Nar protected and restored the expression of ZO-1, Claudin-1, and Occludin at the gene level in lung tissues after LPS induction (Fig. S19). The above results showed that TK-NLP@Nar protected lung barrier integrity, inhibited protein leakage, and ameliorated pulmonary edema.

### 3.7 Biocompatibility evaluation of TK-NLP

**Fig. 7:**
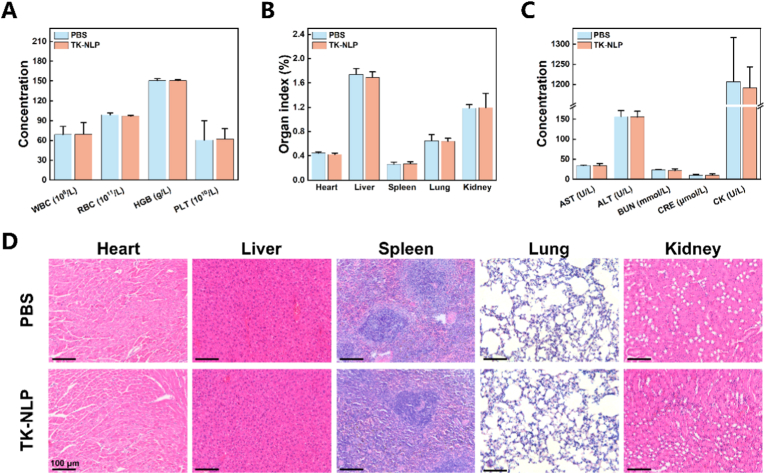
Biocompatibility evaluation of TK-NLP. (**A**) Blood routine (n = 4). (B) Organ index (n = 4). (C) Serum biochemical indices (n = 4). (**D**) H&E-staining of heart, liver, spleen, lung, and kidney. Values shown are mean ± S.D., Scale bar = 100 μm.

We further evaluated the biocompatibility of TK-NLP in healthy mice for 2 days and 14 days. We detected the blood routine, main organs index, H&E staining, and biochemical indices of mice after tail vein injection of PBS (control group), and TK-NLP. The blood routine demonstrated no significant differences among the groups in these indexes of leukocyte, erythrocyte, and platelet, indicated that this nanoparticle injection did not cause an immune response and damage the blood cell *in vivo* (Fig. 7A, S20A). We also recorded the organ index and showed no significant differences between groups (Fig. 7B, S20B). And we displayed serum biochemical indices of the heart (CK), liver (AST, ALT), and kidneys (BUN, CRE), which did not show changes among the groups, indicated these main organs were not damaged (Fig. 7C, S20C). Meanwhile, the H&E staining revealed no apparent pathological structural changes in the heart, liver, spleen, lung, and kidney (Fig. 7D, S20D). These results demonstrated that the TK-NLP has good biocompatibility.

## 4 Conclusion

In summary, we developed a biomimetic drug delivery system with “cruise missile” capabilities. This system leveraged activated neutrophil membranes for precise targeting of injured lung tissue, utilized ROS-triggered mechanisms to rapidly release Nar to ameliorate ALI. This system combines the active targeting capabilities of the neutrophil membrane with ROS-responsive drug release mechanisms. The TK-NLP showed great targeting ability to the injured 2D epithelial cell model and 3D mLO model with/without dynamic cultivation using a microfluidic chip system. In addition, we found that TK-NLP could competitively bind to platelets for inhibiting the formation of PNAs, target and accumulate in the injured lung sites and then respond to the overexpressed ROS to control Nar release, which inhibited the secretion of inflammatory factors, neutrophil infiltration in the lung sites, and protected the integrity of the lung barrier to ameliorate LPS-induced ALI.

## CRediT authorship contribution statement

**Guiquan Liu:** Writing – original draft, Visualization, Validation, Software, Methodology, Investigation, Formal analysis, Data curation, Conceptualization. **Xinting Wang:** Visualization, Validation, Methodology, Investigation, Data curation. **Jia Liu:** Visualization, Data curation. **Haonan Wu:** Software, Data curation. **John Osilama Thomas:** Writing – review & editing. **Yan Zhu:** Writing – review & editing, Supervision, Software, Resources. **Xi Wang:** Writing – review & editing, Visualization, Validation, Methodology, Data curation, Conceptualization. **Jian Yang:** Writing – review & editing, Supervision, Software, Resources, Project administration, Funding acquisition, Formal analysis, Conceptualization.

## Declaration of competing interest

The authors declare that they have no known competing financial interests or personal relationships that could have appeared to influence the work reported in this paper.

## Data Availability

Data will be made available on request.
