# Supplementary material for: A nano-immuno-cruise delivery system encapsulated lipid-integrated bilayer ameliorate acute lung injury by interfering neutrophil infiltration
Source: Mater Today Bio. 2025 Nov 17;35:102563. doi: 10.1016/j.mtbio.2025.102563 (PMC12686634; doi:10.1016/j.mtbio.2025.102563)
Supplement: Multimedia component 1 [file mmc1.docx]

**A nano-immuno-cruise delivery system encapsulated neutrophil membrane and ROS-responsed lipids ameliorate acute lung injury by interfering platelet-neutrophil aggregates formation**

Guiquan Liu ^a,b,c,1^, Xinting Wang ^a,b,c,1^, Jia Liu ^a,b,c^, Haonan Wu ^a,b,c^, John Osilama Thomas ^a,b,c^, Yan Zhu ^a,b,c^, Xi Wang ^a,b,c,*^, Jian Yang ^a,b,c,d,*^

^a^ State Key Laboratory of Chinese Medicine Modernization, Tianjin University of Traditional Chinese Medicine, Tianjin 301617, China

^b^ Institute of Traditional Chinese Medicine, Tianjin University of Traditional Chinese Medicine, Tianjin 301617, China

^c^ Haihe Laboratory of Modern Chinese Medicine, Tianjin 301617, China

^d^ Department of Orthopedics, Binhai New Area Hospital of Traditional Chinese Medicine and the Fourth Affiliated Hospital of Tianjin University of Traditional Chinese Medicine, Tianjin, 301617, China

^1^ Guiquan Liu and Xinting Wang contributed equally to this work.

^*^ **Correspondence:** [wxtjtcm@163.com](mailto:wxtjtcm@163.com) (Xi Wang), [yang.j2017@tjutcm.edu.cn](mailto:yang.j2017@tjutcm.edu.cn) (Jian Yang).

**
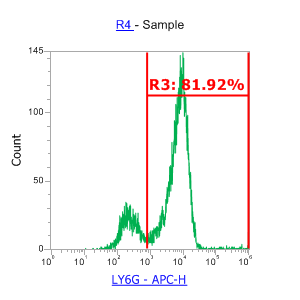
**

**Fig. S1.** The purity of the neutrophils obtained from mouse bone was detected using flow cytometry.


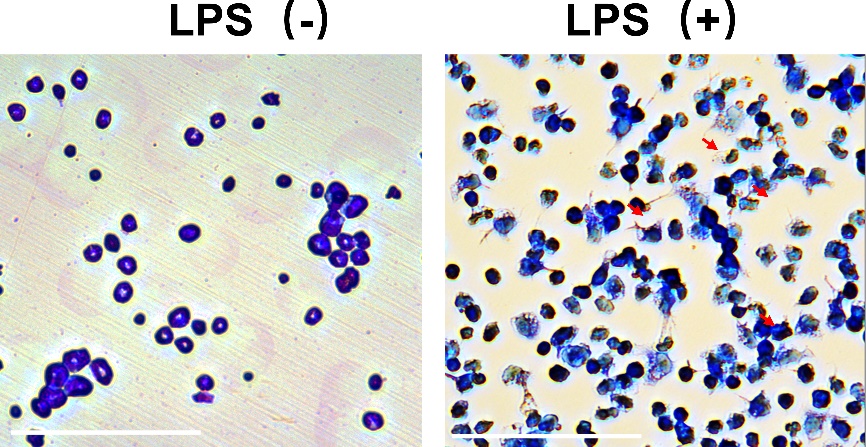


**Fig. S2.** Morphological images of neutrophil (left) and LPS-induced neutrophil (right) after the Wright-Giemsa staining. Scare bar = 100 μm.


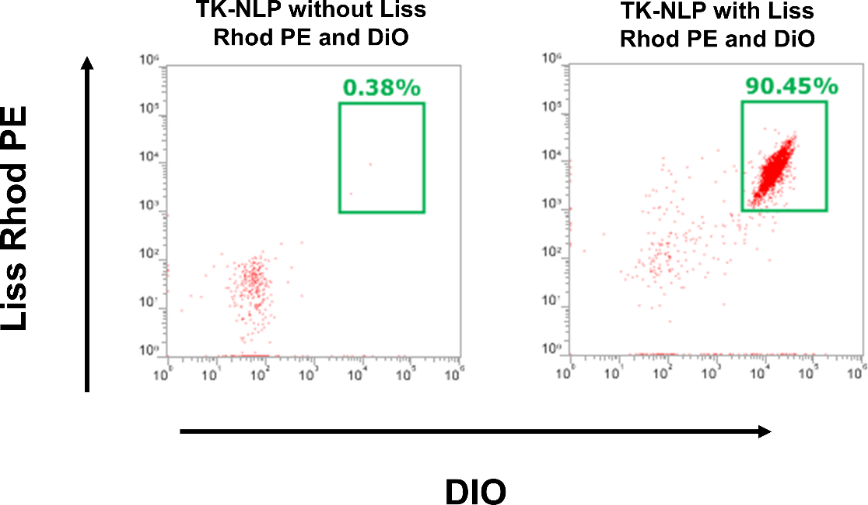


**Fig. S3.** Fusion rate of TK-NLP detected by flow cytometry, the neutrophil membrane was labeled with DiO, the TK-NLP was labeled with Liss Rhod PE.


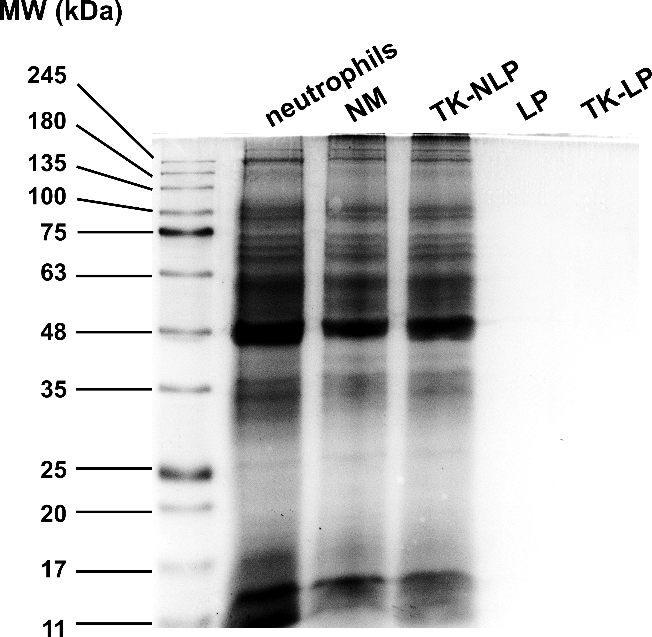


**Fig. S4.** SDS-PAGE protein band analysis of neutrophils, NM, TK-NLP, LP, and TK-LP.


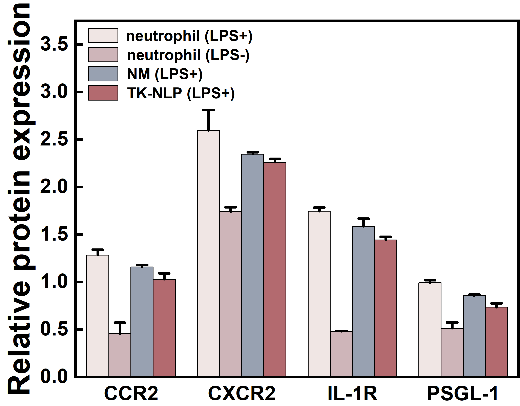


**Fig. S5.** Quantification analysis of membrane receptor proteins including CCR2, IL-1R, PSGL-1 and CXCR2 in neutrophils (LPS+), neutrophils (LPS-), NM (LPS+), and TK-NLP (LPS+) (n = 3). Values shown are mean ± S.D.


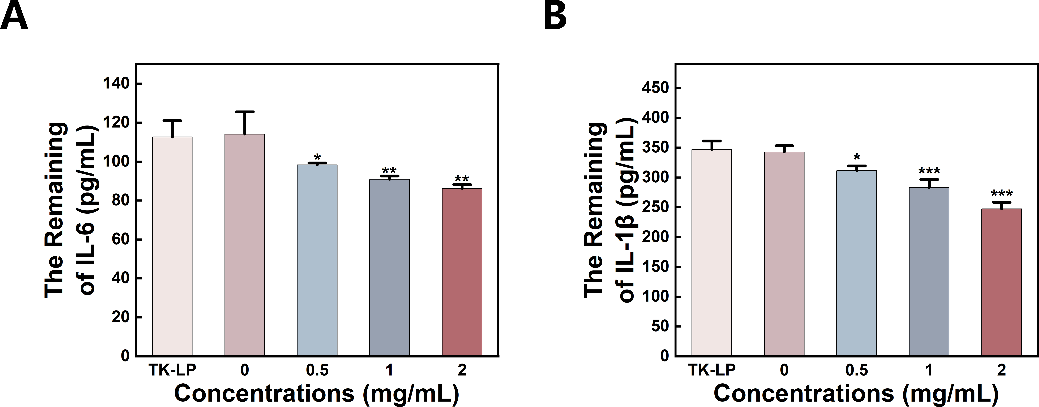


**Fig. S6.** The remained concentrations of (A) IL-6, and (B) IL-1β after co-cultured with 2 mg/mL TK-LP and different concentrations of TK-NLP (n = 3). Values shown are mean ± S.D., **P* < 0.05, ***P* < 0.01, ****P* < 0.001, vs 0 mg/mL.


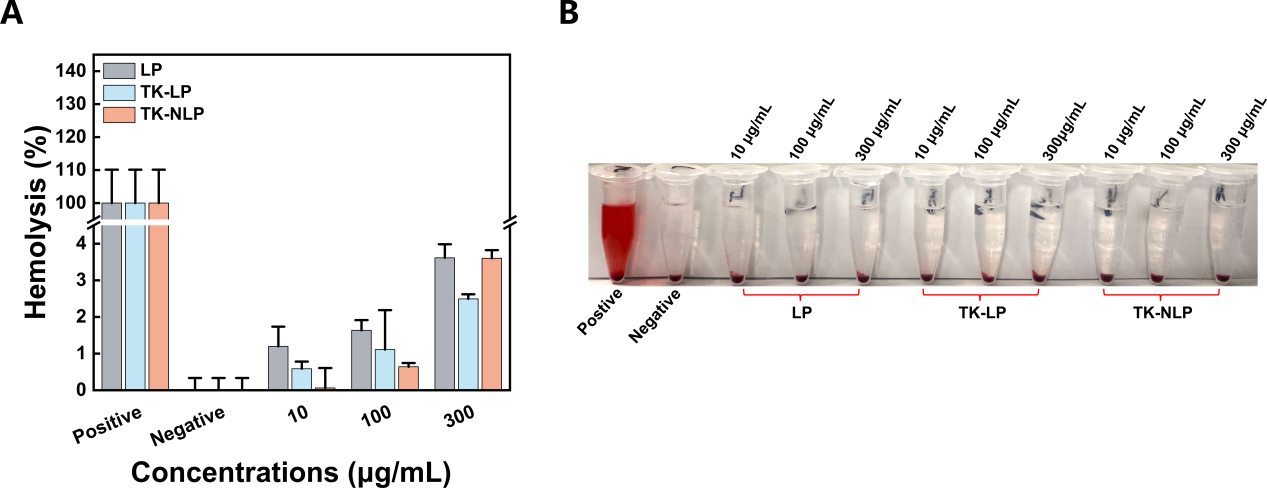


**Fig. S7.** Red blood cell compatibility of TK-NLP. (A) Qualification of hemolysis and (B) images of RBCs after incubated with different concentrations of LP, TK-LP, and TK-NLP (n = 3). Values shown are mean ± S.D.


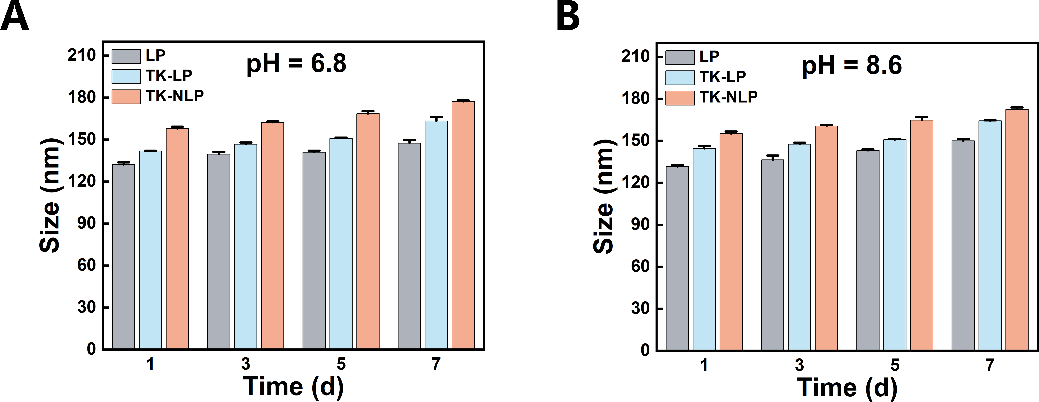


**Fig. S8.** The size changed of LP, TK-LP, and TK-NLP in 7 days were detected using DLS at (**A**) pH=6.8 and (**B**) pH=8.6. Values shown are mean ± S.D.


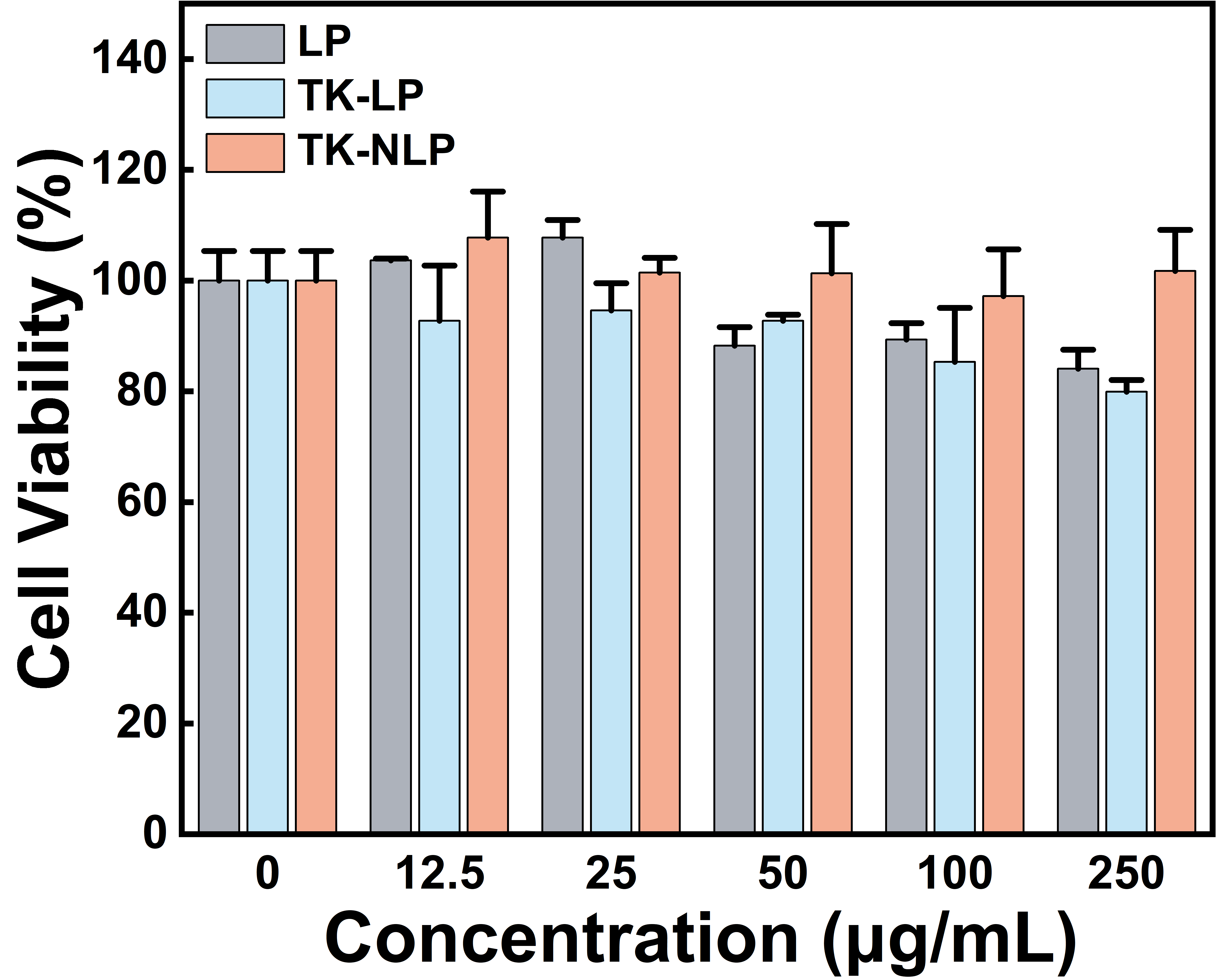


**Fig. S9.** The cell viability of 16HBE cells after incubation with different concentrations of LP, TK-LP, and TK-NLP (n = 5). Values shown are mean ± S.D.


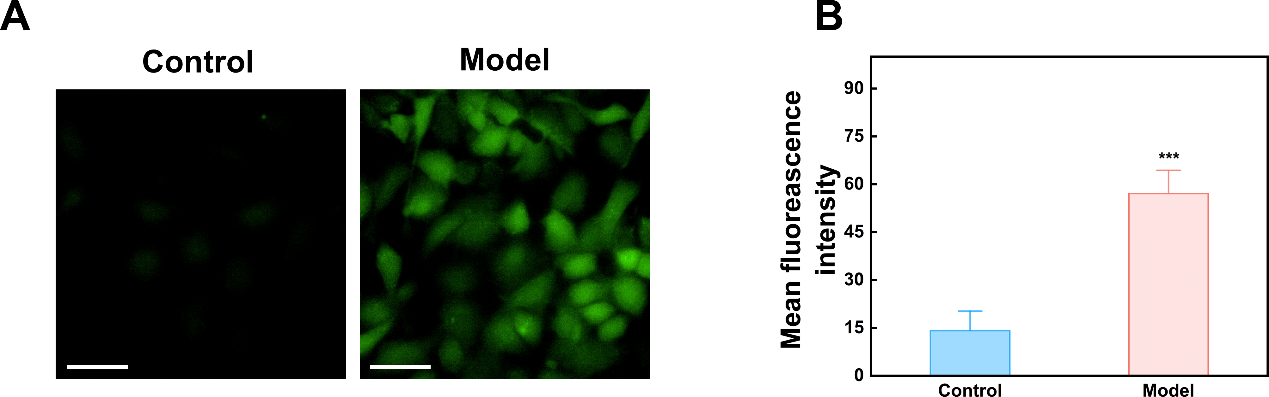


**Fig. S10.** Establishing an *in vitro* model with high expression of ROS using 16HBE. (A) Images of 16HBE before and after LPS-induction. (B) Quantification analysis of mean fluorescence intensity of ROS (n = 5). Values shown are mean ± S.D., ****P* < 0.001. Scare bar = 50 μm.


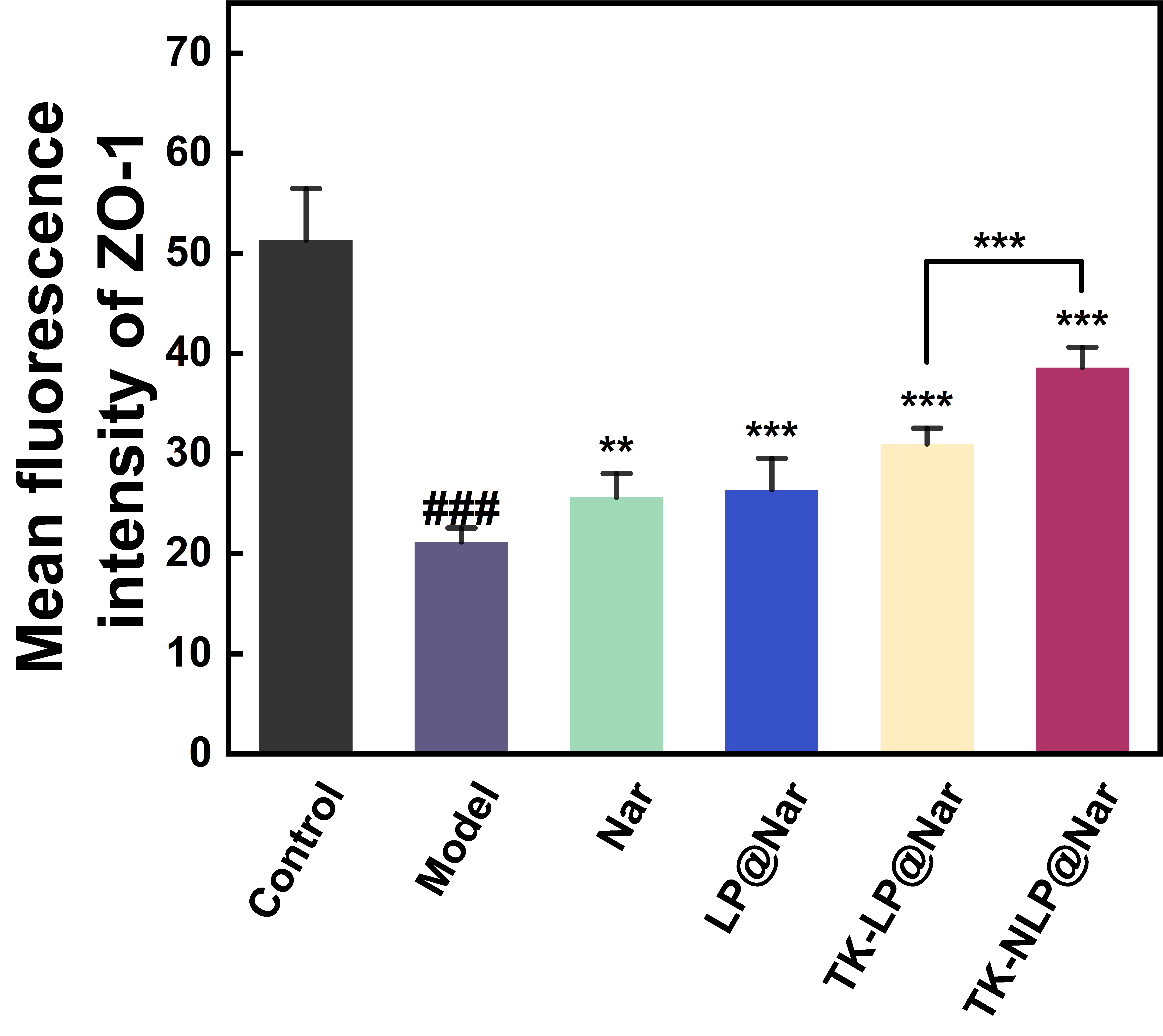


**Fig. S11.** Quantification of the mean fluorescence intensity of ZO-1 in 16HBE (n = 5). Values shown are mean ± S.D., ^###^*P* < 0.001, vs Control group, ***P* < 0.01, ****P* < 0.001, vs Model group.


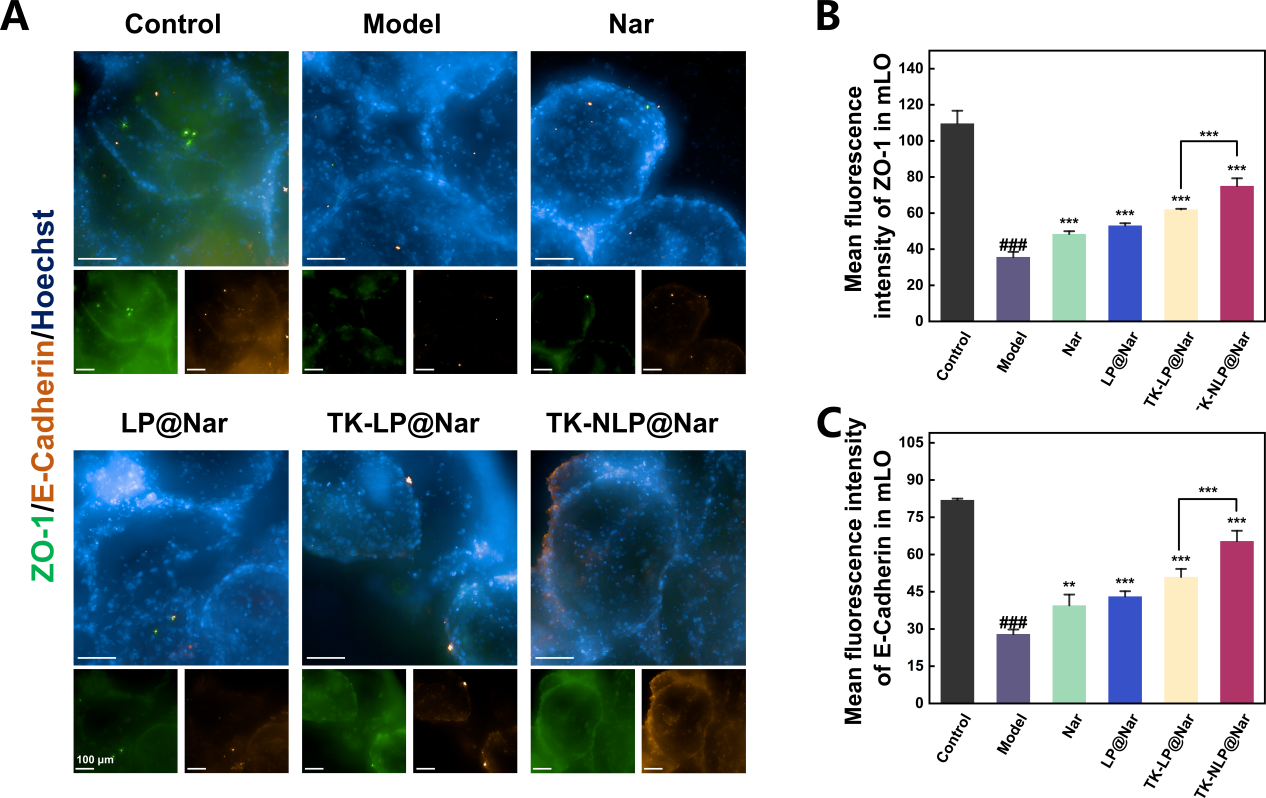


**Fig. S12.** Immunofluorescence staining images of mLO after co-labeled (A) ZO-1 and E-cadherin. The quantification of mean fluorescence intensity of (**B**) ZO-1 and (**C**) E-cadherin (n = 5). Values shown are mean ± S.D., ^###^*P* < 0.001, vs Control group, ***P* < 0.01, ****P* < 0.001, vs Model group. Scare bar = 100 μm.


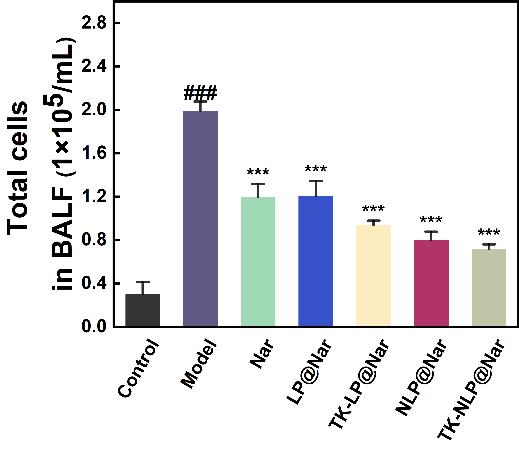


**Fig. S13.** Quantification of the numbers of total cells in BALF (n = 4). Values shown are mean ± S.D., ^###^*P* < 0.001, vs Control group, ****P* < 0.001, vs Model group.


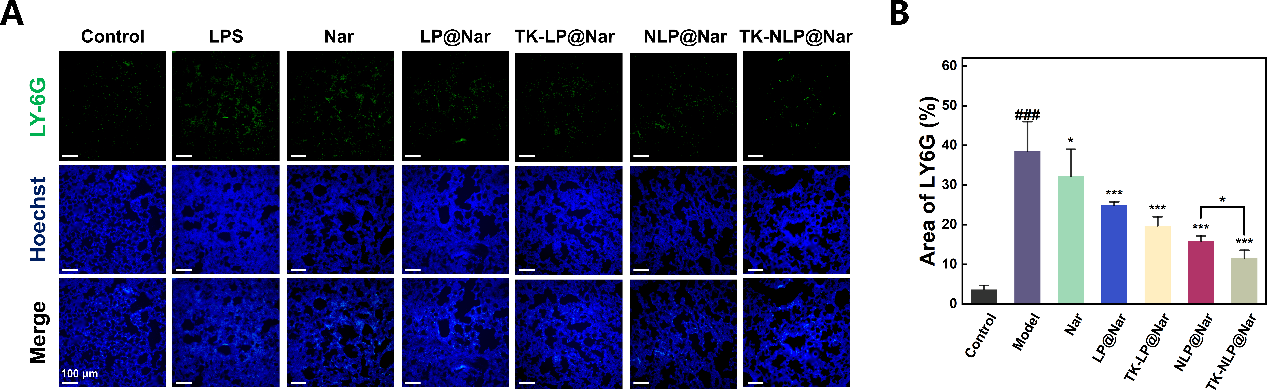

**Fig. S14.** The detection of neutrophil infiltration in lung tissues. (A) The immunofluorescence images and (B) qualification analysis of the area ratios of Ly6G in lung tissue sections (n = 4). Values shown are mean ± S.D., ###*P* < 0.001, vs Control group, **P* < 0.05, ***P* < 0.01, ****P* < 0.001, vs Model group. Scare bar = 100 μm.


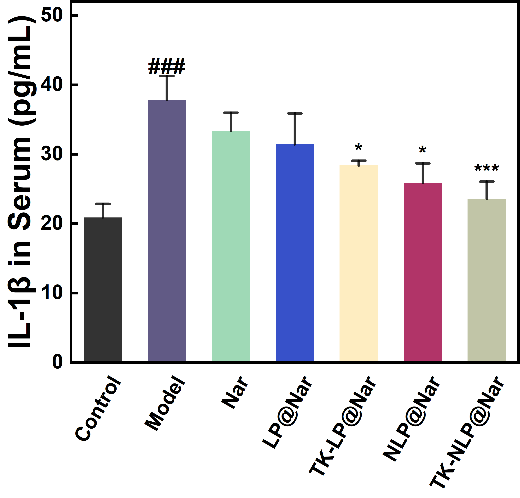


**Fig. S15.** The detection of IL-1β in serum (n = 4). Values shown are mean ± S.D., ###*P* < 0.001, vs Control group, **P* < 0.05, ****P* < 0.001, vs Model group.


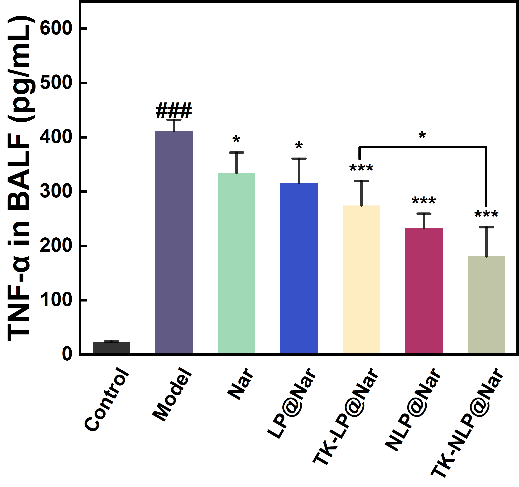


**Fig. S16.** The detection of TNF-α in BALF (n = 4). Values shown are mean ± S.D., ^###^*P* < 0.001, vs Control group, **P* < 0.05, ****P* < 0.001, vs Model group.


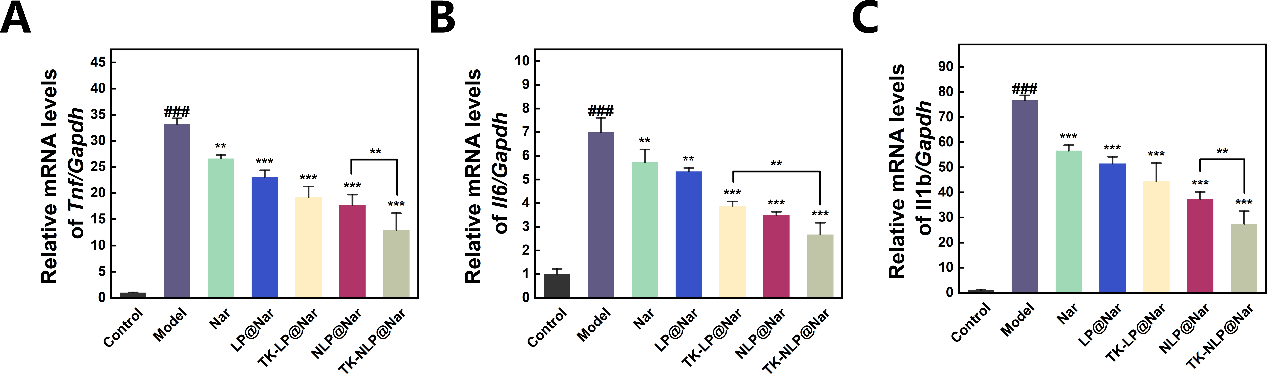


**Fig. S17.** The detection of mRNA levels of (A) TNF-α, (B) IL-6, and (C) IL-1β in lung tissues (n = 4). Values shown are mean ± S.D., ^###^*P* < 0.001, vs Control group, ***P* < 0.01, ****P* < 0.001, vs Model group.

**
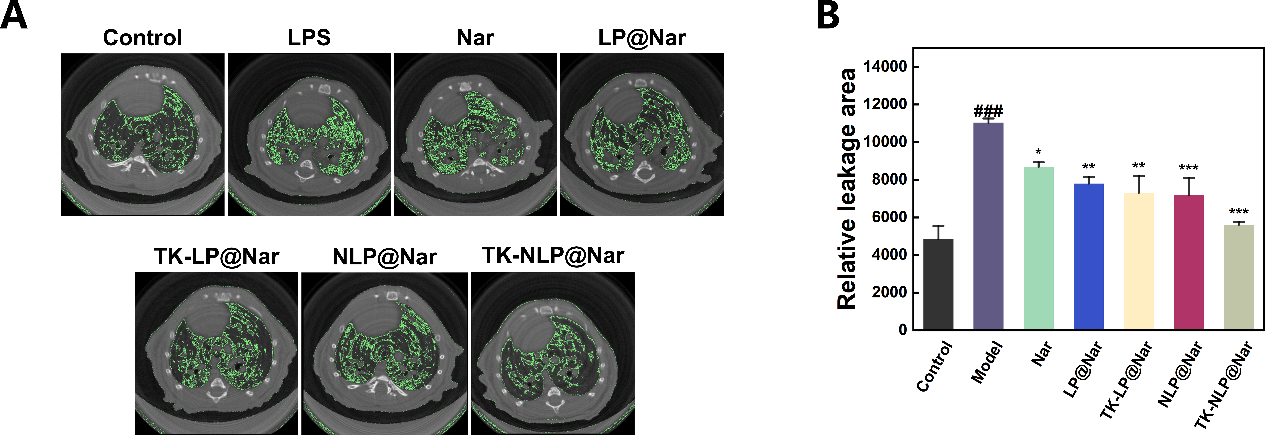
**

**Fig. S18.** (A) Micro-CT images, and (B) quantification of lung injury in mice (n = 4). Micro-CT images were used to analyze the leakage area with green pseudo color. Values shown are mean ± S.D., ###P < 0.001, vs Control group, **P* < 0.05, ***P* < 0.01, ****P* < 0.001, vs Model group.


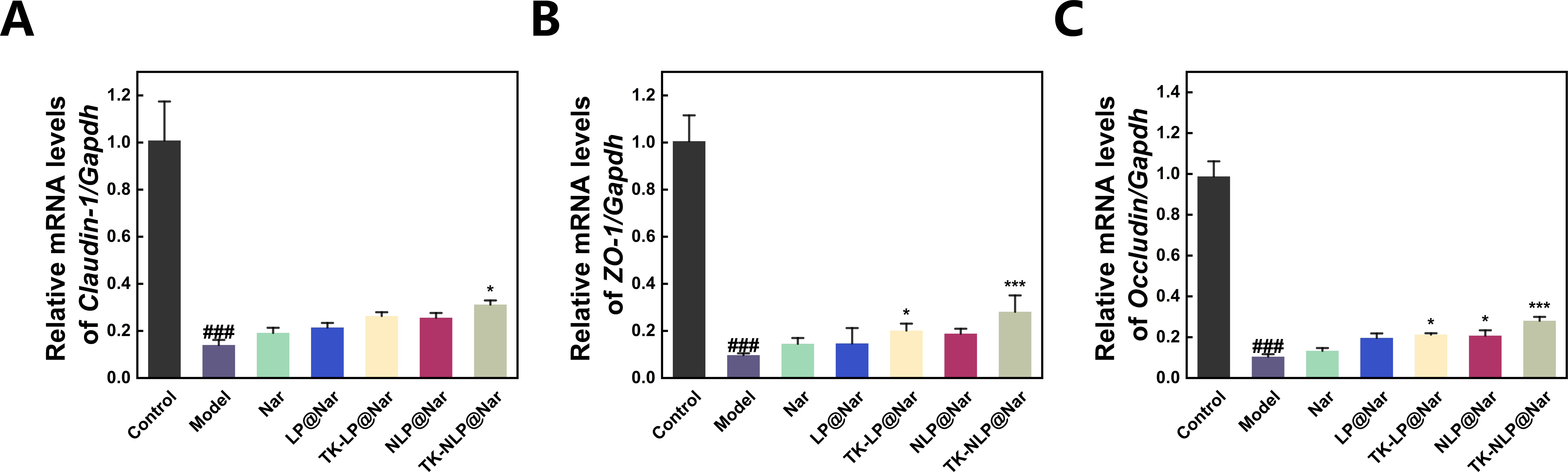


**Fig. S19.** The detection of mRNA levels of (A) Claudin-1, (B) ZO-1, and (C) Occludinin in lung tissues (n = 4). Values shown are mean ± S.D., ^###^*P* < 0.001, vs Control group, **P* < 0.05, ***P* < 0.01, vs Model group.


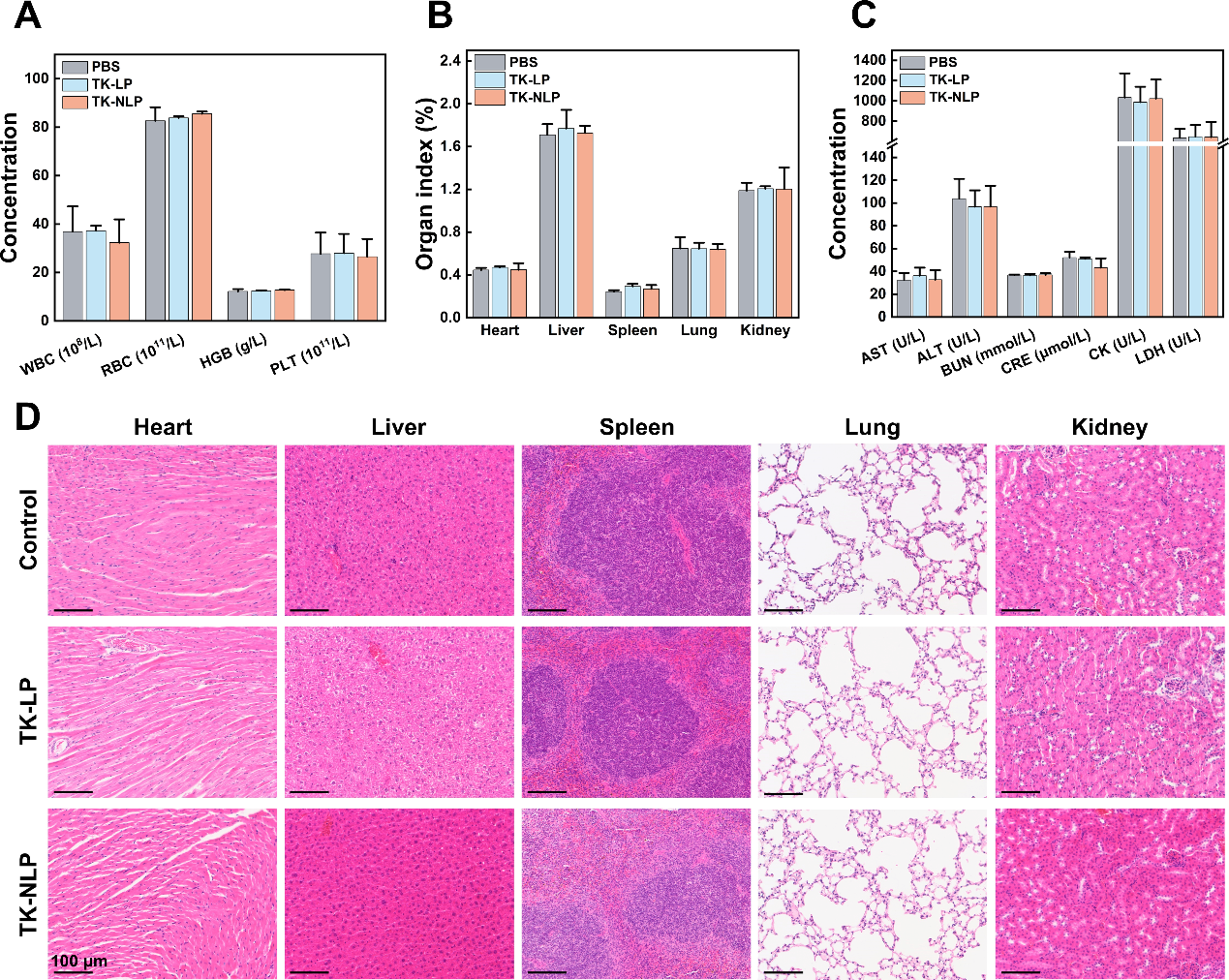


**Fig. S20.** Biocompatibility evaluation of TK-NLP in healthy mice. (A) Blood routine (n = 4). (B) Organ index (n = 4). (C) Serum biochemical indices (n = 4). (D) H&E-staining of heart, liver, spleen, lung, and kidney (n = 4). Values are expressed as mean ± S.D., Scale bar = 100  μm.

**Table S1.** Primer Sequences

| Primer name | Forward Primer (5’-3’) | Reverse Primer (5’-3’) |
| --- | --- | --- |
| *Il6* | TAGTCCTTCCTACCCCAATTTCC | TTGGTCCTTAGCCACTCCTTC |
| *Tnf* | GCACAGAAAGCATGATCCGC | CTGCCACAAGCAGGAATGAG |
| *IL1B* | GCAACTGTTCCTGAACTCAACT | ATCTTTTGGGGTCCGTCAACT |
| *Gapdh* | TGGTGAAGCAGGCATCTGAG | TGCTGTTGAAGTCGCAGGAG |
| *Cldn1* | TGCCCCAGTGGAAGATTTACT | CTTTGCGAAACGCAGGACAT |
| *Ocln* | CCTCTGACCTTGAGTGTGGATGAC | TCCTCTTGCCCTTTCCTGCTTTC |
| *Tjp1* | ACCCGAAACTGATGCTGTGGATAG | GCTGGCTGGCTGTACTGTGAG |
